# Hyperinsulinemia promotes aberrant histone acetylation in triple-negative breast cancer

**DOI:** 10.1186/s13072-019-0290-9

**Published:** 2019-07-17

**Authors:** Parijat Senapati, Hiroyuki Kato, Michael Lee, Amy Leung, Christine Thai, Angelica Sanchez, Emily J. Gallagher, Derek LeRoith, Victoria L. Seewaldt, David K. Ann, Dustin E. Schones

**Affiliations:** 1 Department of Diabetes Complications and Metabolism, Beckman Research Institute, City of Hope, Duarte, CA 91010 USA; 2 Irell & Manella Graduate School of Biological Sciences, City of Hope, Duarte, CA USA; 3 Department of Population Sciences, Beckman Research Institute, City of Hope, Duarte, CA 91010 USA; 4 Division of Endocrinology, Diabetes and Bone Diseases, Icahn School of Medicine at Mount Sinai, New York, NY 10029 USA

**Keywords:** Insulin, Histone acetylation, Hyperinsulinemia, TNBC, Chromatin

## Abstract

**Background:**

Hyperinsulinemia, the presence of excess insulin relative to glucose in the blood, is considered to be a poor prognostic indicator for patients with triple-negative breast cancer (TNBC). mTOR, a downstream effector of insulin, enhances mitochondrial biogenesis and activity, thereby increasing acetyl-CoA precursors. Increased acetyl-CoA can, in turn, be utilized by nuclear acetyltransferases for histone acetylation, a critical feature of genome regulation. While signaling pathways downstream of insulin have been established for sometime, the effect of insulin on chromatin remains unclear. We hypothesized that hyperinsulinemia-induced metabolic changes lead to genome-wide changes in histone acetylation in TNBC.

**Results:**

MDA-MB-231 cells were xenografted into hyperinsulinemic and wild-type mice. Tumors in the hyperinsulinemic mice displayed elevated levels of histone acetylation compared to tumors in normal insulin conditions. We show that insulin treatment in vitro leads to global increase in chromatin-associated histone acetylation, in particular at H3K9, through the PI3K/AKT/mTOR pathway. Genome-wide analyses revealed that most promoter regions have an increase in histone acetylation upon insulin treatment. In addition, insulin induces higher levels of reactive oxygen species and DNA damage foci in cells.

**Conclusions:**

These results demonstrate the impact of hyperinsulinemia on altered gene regulation through chromatin and the importance of targeting hyperinsulinemia-induced processes that lead to chromatin dysfunction in TNBC.

**Electronic supplementary material:**

The online version of this article (10.1186/s13072-019-0290-9) contains supplementary material, which is available to authorized users.

## Background

Metabolic syndrome is a collection of risk factors for cardiovascular disease and diabetes, and several types of cancer [1]. Multiple factors common to metabolic syndrome, including hyperinsulinemia, hyperglycemia, hyperlipidemia as well as altered adiponectin and leptin levels, can promote tumor growth and progression [2]. Hyperinsulinemia alone, even in the absence of obesity and type 2 diabetes (T2D), is associated with increased incidence [3–6] and adverse prognosis [7, 8] for breast cancer. Triple-negative breast cancer (TNBC) is a clinically aggressive subtype of breast cancer that does not express estrogen receptor (ER), progesterone receptor (PR) or human epidermal growth factor receptor 2 (HER2) [9]. An increasing body of literature suggests that metabolic syndrome is an additional risk factor for developing TNBC in premenopausal women [10].

The insulin signaling pathway has many effectors that can promote cancer development. Insulin can stimulate tumor cell survival and proliferation by signaling through the insulin receptor (IR) [11, 12]. High circulating insulin levels can also enhance the available pool of insulin-like growth factor (IGF-I) by decreasing the expression of IGF-binding protein 1 (IGFBP1) [13]. IGF-I is a potent mitogen that can induce cell proliferation in several cell types [14]. Furthermore, insulin binding to IR leads to downstream activation of PI3K/AKT and MAPK signaling pathways [15]. The PI3K/AKT pathway has oncogenic properties and in addition induces mTOR signaling to promote cell growth [16, 17]. mTOR signaling can also stimulate mitochondrial biogenesis and activity, which increases TCA cycle utilization and ATP production through increased rates of oxidative phosphorylation [18].

Multiple lines of evidence indicate that hyperinsulinemia in patients is an important factor in breast cancer biology. Increased IR expression and the presence of phosphorylated IR/IGF-IR in breast cancer are associated with poor prognosis and decreased survival [19, 20]. Breast cancers frequently show deregulation of the PI3K/AKT and mTOR pathway [21–24]. Moreover, activation of AKT/mTOR signaling is associated with poor prognosis in TNBC [25]. Since insulin can activate the PI3K/AKT/mTOR pathway, it is predicted that hyperinsulinemia, in the absence of other metabolic dysfunctions, may drive the aggressive biology of TNBCs in insulin-resistant women. Indeed, in a mouse model of hyperinsulinemia without confounding factors such as obesity, hyperglycemia or hyperlipidemia [26], endogenous hyperinsulinemia increases mammary tumor growth as well as metastases [27] by signaling primarily through the IR [26, 28].

It is becoming apparent that metabolic products including methyl- and acetyl-donors influence the rate of modifications to DNA and histones. Acetyl-coenzyme A (acetyl-CoA) is one of these important products. In cancer cells, metabolic reprogramming leads to increased utilization of glucose through glycolysis and the TCA cycle. In proliferating cancer cells, citrate from the TCA cycle is converted to acetyl-CoA that is utilized for lipid production [29]. In the nucleus, acetyl-CoA is a substrate for histone acetyltransferases, such as p300 and CBP, to modify histone tails [30, 31]. Histone acetylation is shown to be an important modification regulating gene expression by increasing the accessibility of chromatin [30, 31]. Thus, acetyl-CoA is a key metabolite linking metabolism, signaling and the epigenome.

Many studies, as we have discussed above, have indicated a role for hyperinsulinemia in breast cancer. However, it is unclear how this may directly impact TNBC cells. Tumor growth and malignant transformation are often associated with deregulation of histone acetylation [31], suggesting that maintaining appropriate levels of this posttranslational modification and thereby chromatin accessibility is important to prevent oncogenesis. Physiological levels of acetyl-CoA in cells are comparable to the Km values of histone acetyltransferases. Therefore, fluctuations in acetyl-CoA levels can translate into altered enzymatic activity and histone acetylation [32]. We therefore hypothesize that elevated insulin drives increased production of acetyl-CoA, which leads to increase in histone acetylation to impact nuclear processes.

We report here an investigation into the effects of hyperinsulinemia on chromatin acetylation. We find that insulin induces global increases in histone acetylation in vivo, in MDA-MB-231 cells xenografted into a hyperinsulinemic mouse model, as well as in vitro. Insulin-induced histone acetylation occurs through the PI3K/AKT/mTOR pathway and occurs on chromatin-associated histones. Quantitative ChIP-seq analyses (ChIP-Rx) showed that gene promoters exhibit the greatest increases in histone acetylation. We furthermore provide evidence that insulin triggers an increase in mitochondrial biogenesis and bioenergetics, which when blocked by metformin attenuates the increase in H3K9 acetylation. Finally, insulin induces DNA damage in cells through enhanced reactive oxygen species (ROS) production and increased chromatin accessibility. These findings suggest that hyperinsulinemia leads to altered cell metabolism that influences chromatin acetylation in tumor cells, thereby influencing gene expression across the genome.

## Results

### Hyperinsulinemia increases histone acetylation in vivo and in vitro

**Fig. 1:**
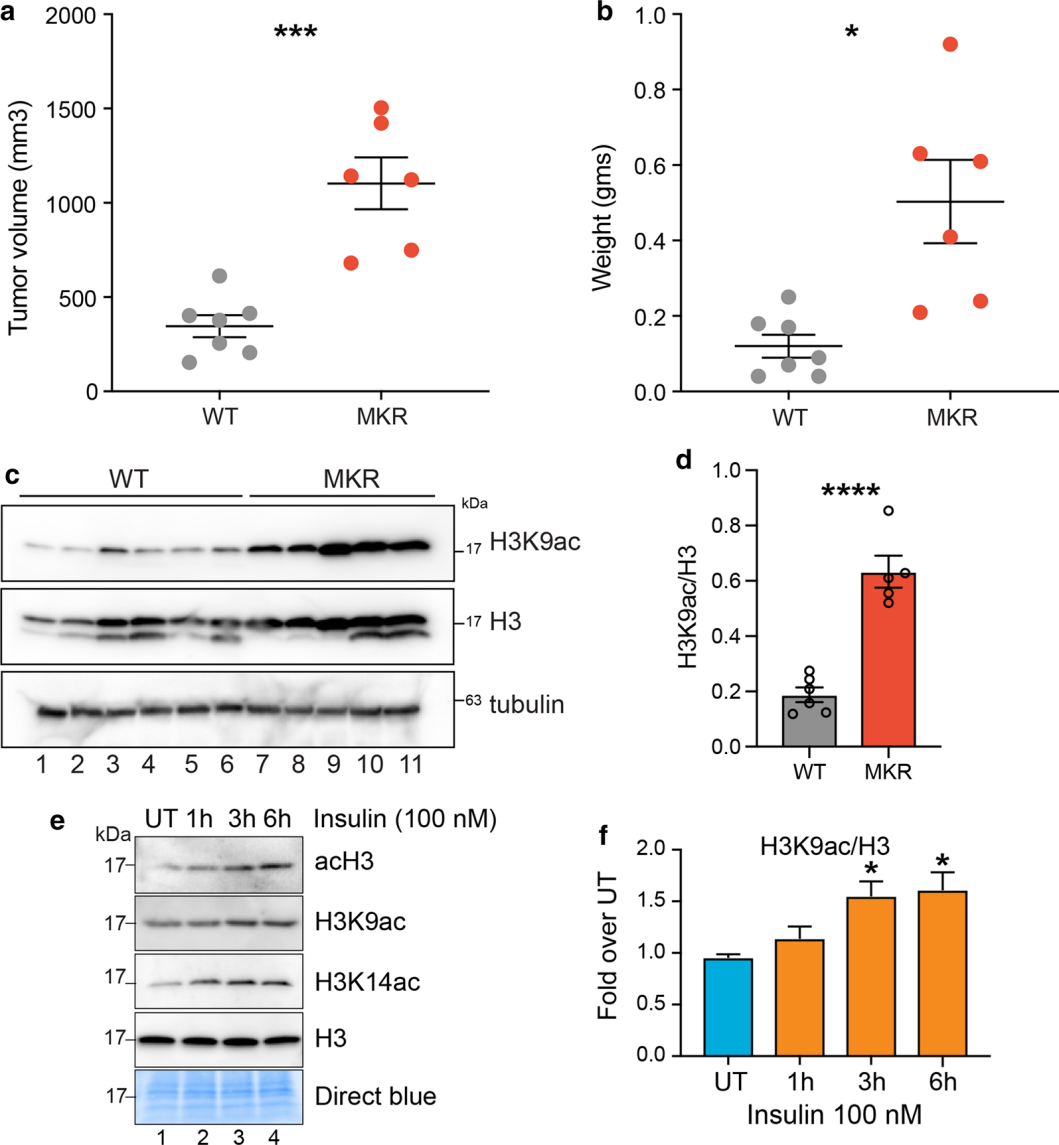
Hyperinsulinemia in MKR mice and in vitro promotes an increase in histone acetylation. **a** Tumor volume (mm^3^) and **b** weight are shown for tumors from Rag/WT (WT) and Rag/MKR (MKR) mice. Significance was calculated using unpaired Student’s *t* test. *p < 0.05, ***p < 0.001. **c** Western blot analysis showing the total levels of H3K9ac and H3, tubulin controls, in WT and MKR tumors. **d** Densitometric quantification of H3K9ac/H3 western blot signals in **c**. Values are Mean + SEM; *n* = 6 for Rag/WT and n = 5 for Rag/MKR lysates. Significance was calculated using unpaired Student’s *t* test. ****p < 0.0001. **e** Western blot analysis using the indicated antibodies in MDA-MB-231 (TNBC cell line) cell lysates treated with insulin (100 nM) for 1 h, 3 h or 6 h. **f** Densitometric quantification of H3K9ac/H3 western blot signals in (**e**). Data are represented as fold change over UT. Values are Mean + SEM; *n* = 3. Statistical significance was calculated using one-way ANOVA, Dunnett’s multiple comparisons test. *p < 0.05

To begin to investigate the effect of elevated insulin levels on histone acetylation and nuclear gene regulation, we utilized an immunodeficient hyperinsulinemic mouse model, *Rag1*^−/−^/MKR^+/+^ [33]. MKR mice harbor a dominant negative mutation in the IGF-IR expressed specifically in the skeletal muscle [26]. The female *Rag1*^−/−^/MKR^+/+^ mice develop hyperinsulinemia but do not exhibit obesity, hyperglycemia or dyslipidemia [26]. Orthotopic tumor xenografts were performed in *Rag1*^−/−^/MKR^+/+^ (Rag/MKR) mice and *Rag1*^−/−^ (Rag/WT) female mice using MDA-MB-231 cells, as previously described [34]. Tumors derived from the Rag/MKR mice were significantly larger and weighed more than those derived from the MKR tumors, as previously described [34] (Fig. 1a, b). To investigate whether tumors from hyperinsulinemic mice showed increased histone acetylation, we performed western blot analysis on tumor protein extracts from Rag/WT or Rag/MKR mice. Results show significantly increased histone acetylation in tumors from Rag/MKR mice (Fig. 1c, d). To directly investigate the effect of insulin on chromatin, we treated MDA-MB-231 cells in vitro with 100 nM insulin for different durations (1 h, 3 h and 6 h) and assayed the levels of histone acetylation via western blot. We observed an increase in total histone H3 acetylation levels (acH3) after 3 h of insulin treatment (Fig. 1e, f). This increase was more pronounced for specific residues such as H3K9 and H3K14 (Fig. 1e, f). These results indicate that hyperinsulinemia enhances histone acetylation in MDA-MB-231 cells in vitro and tumors in vivo. We further assessed global histone acetylation levels in one additional TNBC cell line, MDA-MB-436, as well as several other non-TNBC cell lines: T47D, AU565 and HCC1954 (Additional file 1: Figure S1A–D). While levels of histone acetylation increased in MDA-MB-436 cells after insulin treatment, histone acetylation did not increase in T47D, AU565 or HCC1954 cells, suggesting that the increase in H3K9ac levels by insulin is specific to TNBC cell lines.

### Histone acetylation induced by insulin is dependent on the PI3K/AKT/mTOR pathway

**Fig. 2:**
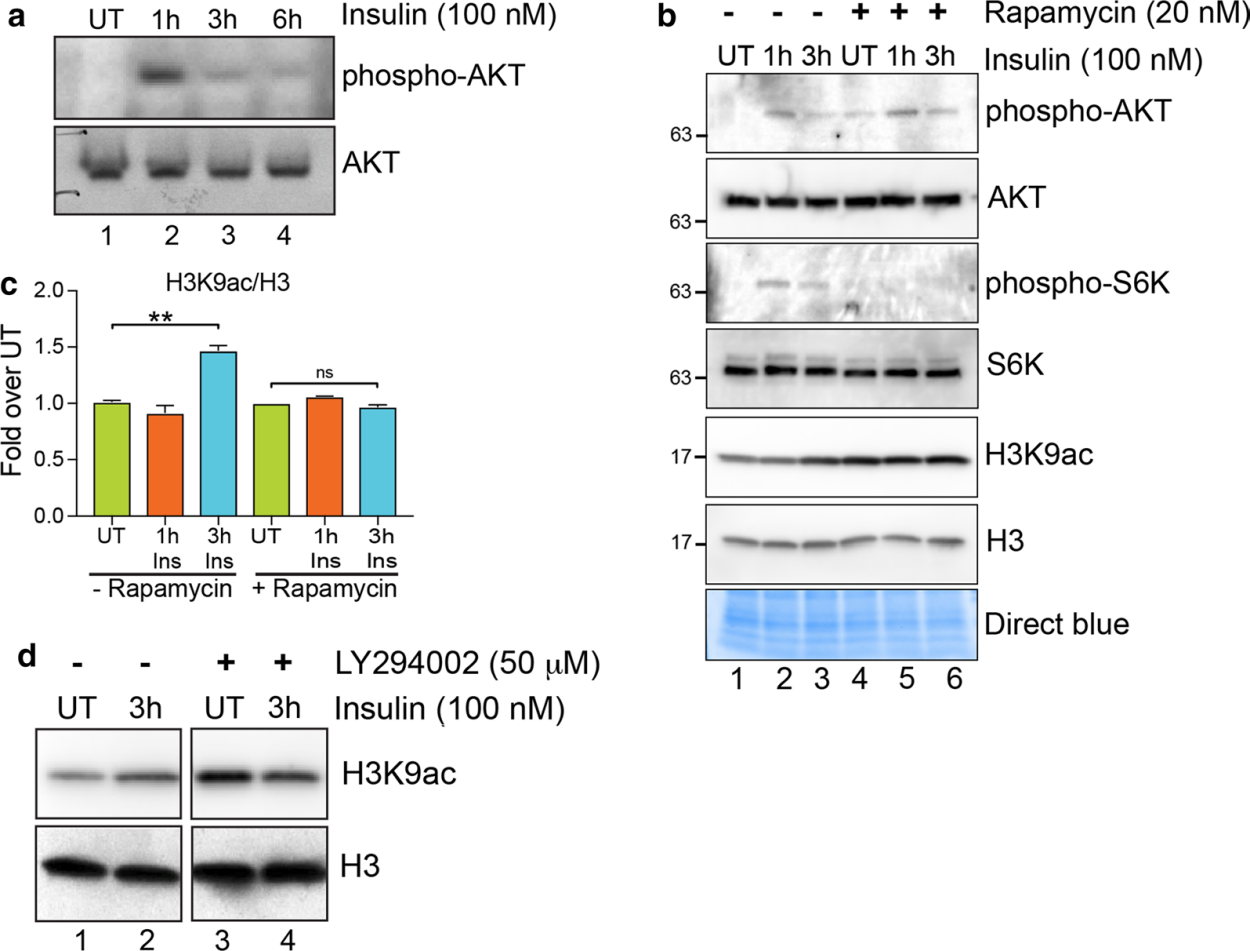
Loss of mTOR and PI3K signaling blocks H3K9ac induced by insulin. **a** Western blot analysis using the indicated antibodies in MDA-MB-231 cell lysates treated with insulin (100 nM) for 1 h, 3 h or 6 h. **b** Western blot analysis using the indicated antibodies in MDA-MB-231 cells pretreated (lanes 4–6) or not (lanes 1–3) with 20 nM mTOR inhibitor rapamycin (1 h) followed by insulin treatment for 3 h. **c** Densitometric quantification of H3K9ac/H3 western blot signals in (**b**). Data are represented as fold change over respective UT. Values are Mean + SEM; *n* = 3. Statistical significance was calculated using one-way ANOVA, Tukey’s multiple comparisons test. **p < 0.01. **d** Western blot analysis using the indicated antibodies in MDA-MB-231 cells pretreated (lanes 3 and 4) or not (lanes 1 and 2) with 50-µM PI3K inhibitor LY294002 (1 h) followed by insulin treatment for 3 h

To confirm that the PI3K/AKT pathway was activated by insulin, we assessed the levels of phospho-AKT by western blot using a phospho-AKT antibody. Phospho-AKT levels were induced at higher levels after 1 h of insulin treatment, after which the signal was attenuated, as expected (Fig. 2a). To assess the role of PI3K/AKT/mTOR pathway activation in increasing histone acetylation levels, we inhibited the action of mTOR and PI3K kinases using specific small molecule inhibitors. We pretreated MDA-MB-231 cells with an mTOR inhibitor, rapamycin, and PI3K inhibitor, LY294002, followed by insulin treatment. AKT and p70 S6 kinase (S6K) (an mTOR kinase substrate) was induced by insulin treatment in rapamycin untreated cells (Fig. 2b, lanes 1 and 2, 3). Rapamycin pre-treatment, however, inhibited the phosphorylation of S6K without affecting AKT phosphorylation, confirming that rapamycin indeed inhibited mTOR kinase activity (Fig. 2b, lanes 4 and 5, 6). Both mTOR inhibition and PI3K inhibition, by rapamycin and LY294002 treatment, respectively, suppressed the H3K9ac increase induced by insulin (Fig. 2b–d). These results show that insulin induces global histone acetylation by activating the PI3K/AKT/mTOR pathway.

### Insulin induces H3K9 acetylation on promoter regions

**Fig. 3:**
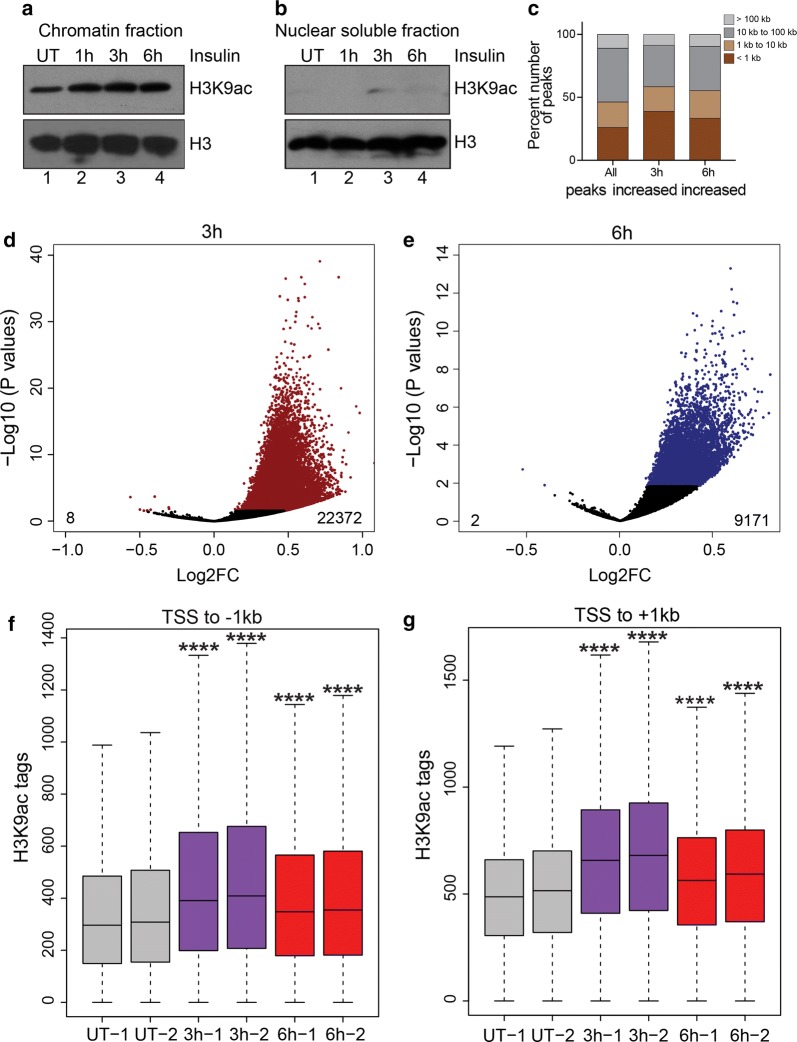
Insulin induces H3K9 acetylation on promoter regions. **a** Western blot analysis for H3K9ac in chromatin fraction or **b** nuclear soluble fraction (right panel) extracted from insulin-treated cells. **c** Stacked bars showing the distribution of H3K9ac peaks categorized by distance to nearest transcription start site (TSS). **d** Volcano plot showing the 22,372 peaks that increased and 8 peaks that decreased H3K9ac acetylation after 3 h insulin treatment. **e** Volcano plot showing the 9171 peaks that increased and 2 peaks that decreased H3K9ac acetylation after 6 h insulin treatment. **f** Box plots showing the distribution of peak scores at − 1 kb to TSS regions of significantly increased H3K9ac peaks. **g** Box plots showing the distribution of peak scores at TSS to + 1-kb regions of significantly increased H3K9ac peaks. Significance was calculated using Kruskal–Wallis test followed by Dunn’s multiple comparisons test. Adjusted *p* values were calculated using Benjamini–Hochberg method. ****p < 0.0001

To confirm that the insulin-induced histone H3 acetylation is chromatin bound and not on newly synthesized or free histones, we performed chromatin fractionation after insulin treatment followed by western blot analyses. Results from these experiments demonstrated that insulin-induced H3K9ac was exclusively chromatin bound (Fig. 3a, b). To characterize the genomic loci associated with increased histone acetylation after insulin treatment, we performed quantitative ChIP-seq analyses [35] (see Additional file 1: Supplementary materials and methods). By performing spike normalization (see Additional file 1: Supplementary materials and methods, Figure S2A–F), we observed global increases in H3K9ac levels on peaks in 3 h and 6 h insulin-treated cells (Additional file 1: Figure S2A–F).

To identify the genomic loci associated with enhanced histone acetylation, we annotated the H3K9ac peaks based on distance to the nearest RefSeq-annotated TSS. Almost half of H3K9ac peaks were promoter proximal (~ 26% within 1 kb and an additional ~ 20% between 1 and 10 kb of nearest TSS) (Fig. 3c). We used DESeq2 [36] to identify peaks that were significantly increased (adjusted *p *< 0.05) in both 3 h and 6 h insulin-treated cells compared to UT; 22,372 and 9,171 peaks had significant increase in H3K9ac at the 3 h and 6 h treatment time points, respectively (Fig. 3d, e). Of the significantly increased peaks, approximately 58% and 55% of the peaks were promoter proximal (Fig. 3c) in 3 h and 6 h samples, respectively, indicating that increases in H3K9ac were predominantly localized at promoter regions and could potentially influence gene expression. Heat maps of H3K9ac at ± 2 kb around annotated start sites of transcripts further confirmed the increase in H3K9ac signal at promoter regions in insulin-treated cells. There was a higher enrichment of H3K9ac signals at promoters in 3 h compared to 6 h treated cells (Additional file 1: Figure S3). This was also evident at TSS ± 1 kb regions of genes with increased H3K9ac (Fig. 3f, g). These results show that insulin induces genome-wide increase in H3K9ac at promoter regions of genes and thereby could be involved in transcriptional regulation.

### Insulin induces H3K9ac on promoters of insulin-induced genes

**Fig. 4:**
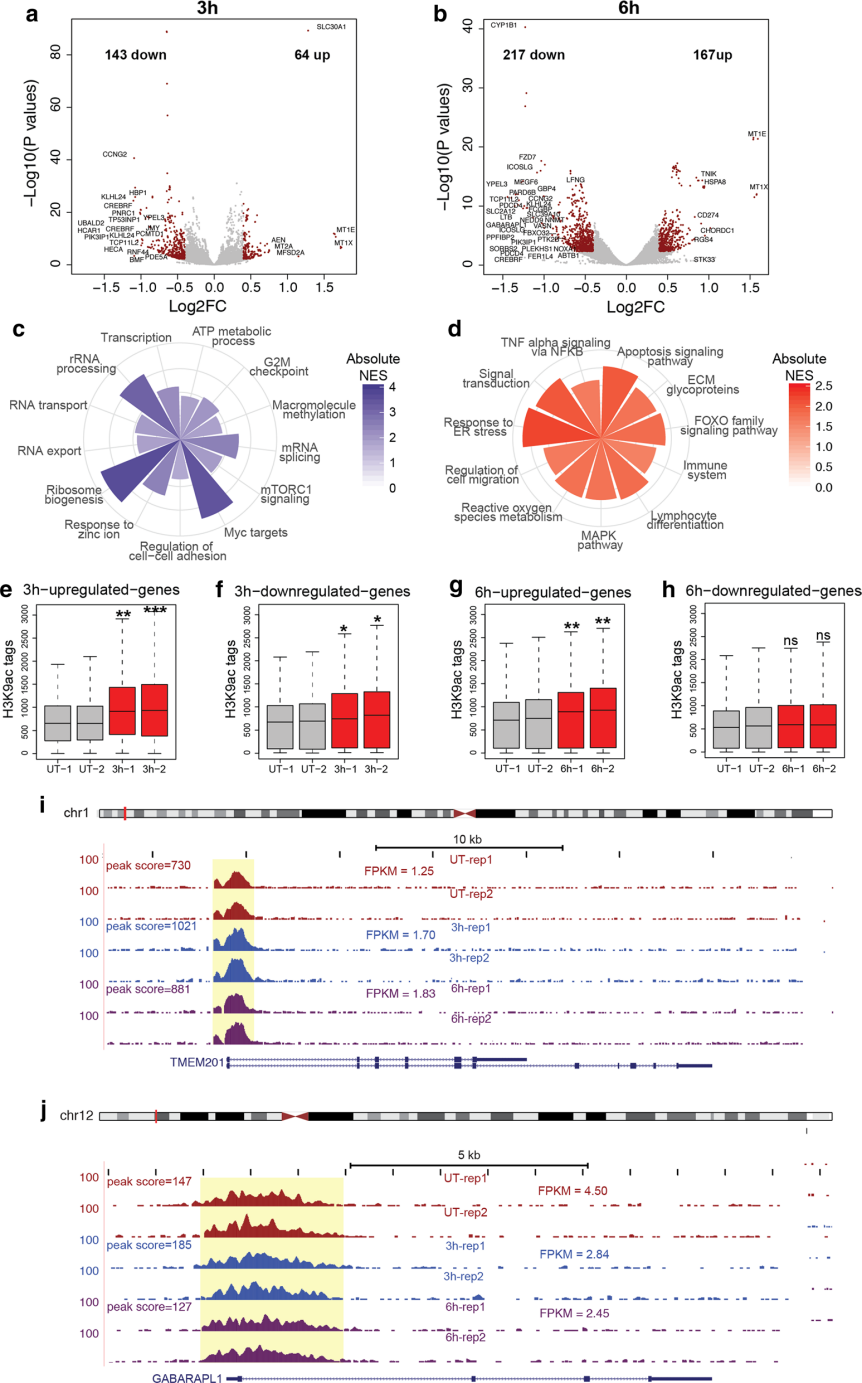
Insulin induces specific increases in H3K9ac acetylation on promoters of insulin-induced genes. **a** Volcano plots showing the genes differentially expressed after 3 h and **b** 6 h insulin treatment. Differentially expressed genes are highlighted in red. **c** Gene sets enriched in insulin-upregulated genes and **d** insulin-downregulated genes. Absolute value of Normalized enrichment score (NES) from Gene Set Enrichment Analysis (GSEA) is shown. *p* < 0.05. **e**–**h** Box plots showing the normalized H3K9ac signal at promoters (TSS ± 1 kb) of genes upregulated and downregulated after 3 h or 6 h of insulin treatment as indicated. Significance was calculated using Kruskal–Wallis test followed by Dunn’s multiple comparisons test. Adjusted *p* values were calculated using Benjamini–Hochberg method. *p < 0.05, **p < 0.01, ***p < 0.001. **i** Genome browser screen shots showing the H3K9ac signal at *TMEM201* (upregulated gene) and **j** *GABARAPL1* (downregulated gene). Expression values (FPKM) an peak scores are shown

To further test whether the increase in H3K9ac enrichment correlates with gene expression changes induced by insulin, we performed transcriptome analysis using RNA sequencing (RNA-seq) in untreated (UT) MDA-MB-231 cells or treated for 3 h and 6 h with insulin. We quantified changes in gene expression after 3 h and 6 h of insulin treatment from RNA-seq data using DESeq2 [36]; 207 and 384 genes exhibited significantly altered expression in 3 h and 6 h insulin-treated cells, respectively (Fig. 4a, b). Genes altered by insulin treatment showed enrichment of specific signaling pathways. Metabolic pathways required for cellular growth such as ribosome biogenesis, transcription, splicing as well as known insulin regulated pathways such as ATP production and mTOR signaling were induced by insulin (Fig. 4c). Genes downregulated by insulin include FOXO signaling genes as well as apoptosis-inducing genes. Interestingly, insulin treatment also downregulated genes involved in reactive oxygen species (ROS) metabolism or scavenging as well as immune cell migration and activation (Fig. 4d). Moreover, insulin upregulated several MYC (c-Myc) target genes and genes related to zinc ion homeostasis in cells (Fig. 4c). These results indicate that insulin signaling alters specific gene expression programs that aid cell growth and proliferation while also suppressing apoptosis.

To test whether genes altered by insulin had concordant changes in H3K9ac enrichment, we compared the H3K9ac signals on the promoter regions (TSS ± 1 kb) of genes upregulated and downregulated by insulin. Upregulated genes showed a larger increase in H3K9ac enrichment induced by insulin (Fig. 4e, g) at 3 h and 6 h, respectively. However, genes downregulated at 3 h also showed a modest but significant increase in H3K9ac enrichment at their promoters (Fig. 4f). Interestingly, genes downregulated after 6 h insulin treatment did not show any significant enrichment in H3K9ac signals (Fig. 4h). These results indicate that there is a genome-wide increase in H3K9ac signal at all expressed genes after 3 h insulin treatment. However, at 6 h the increase in H3K9ac signal is more specific to upregulated genes. Representative examples show the increase in H3K9ac enrichment on the upregulated gene *TMEM201* (Fig. 4i) and no change in H3K9ac signal on the promoter of *GABARAPL1* (Fig. 4j), a downregulated gene. Moreover, we validated the expression of a number of upregulated genes that show increase in H3K9ac upon insulin treatment by RT-qPCR (Additional file 1: Figure S4). These results indicate that the gene expression changes observed after insulin treatment are specific and are likely induced by signal-dependent transcription factors. Genome-wide increase in H3K9ac at promoters may facilitate increased chromatin accessibility at regulatory regions, however, is not sufficient to alter transcription which depends on recruitment of transcription factors, co-activators and RNA polymerase II.

### Transcription factor NRF1 is involved in insulin-mediated gene expression changes and histone acetylation

**Fig. 5:**
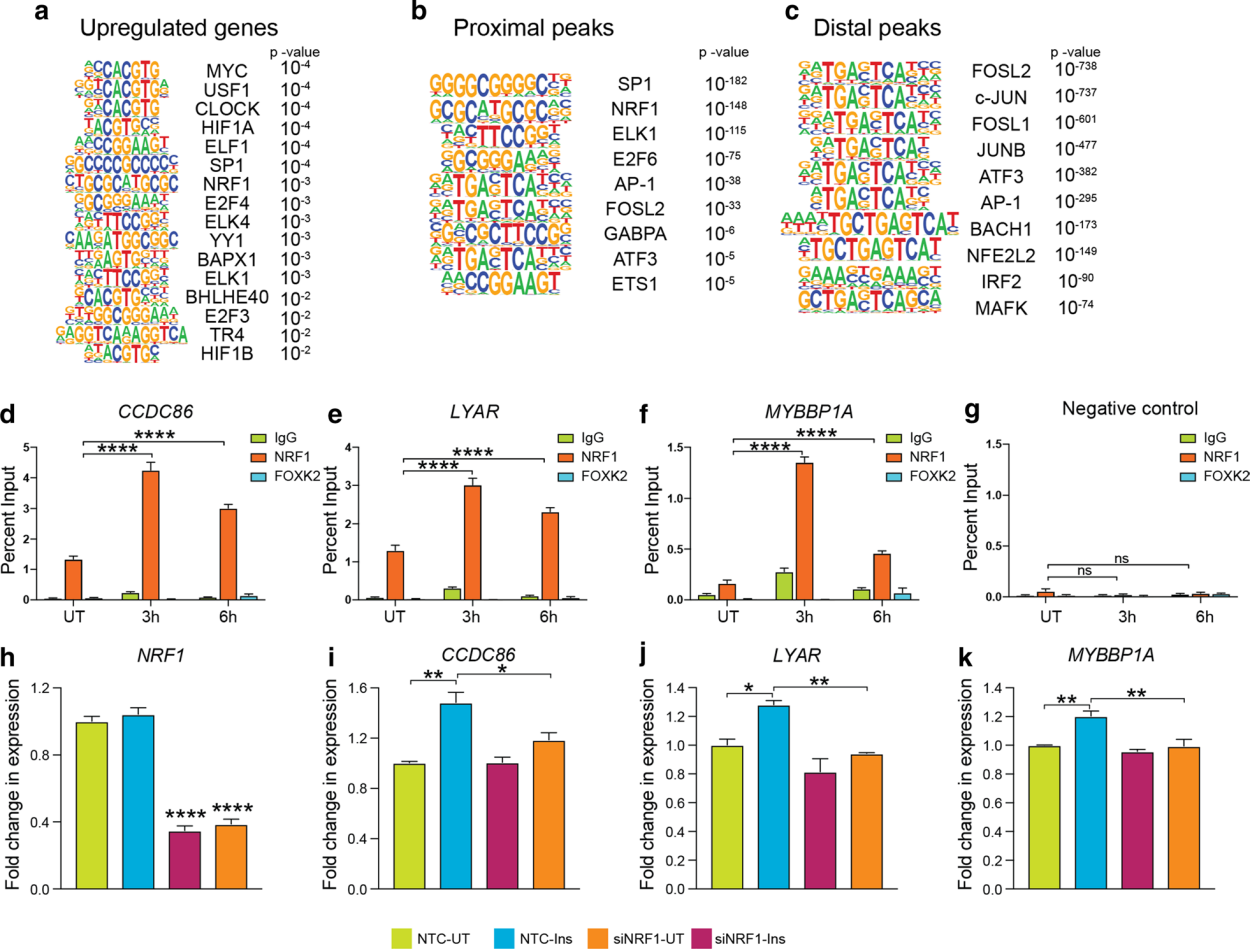
Transcription factors NRF1 is involved in insulin-mediated gene expression changes and chromatin remodeling. **a** Transcription factor-binding motifs enriched in promoter regions (TSS ± 1 kb) of upregulated genes. **b** Transcription factor-binding motifs enriched in promoter proximal and **c** distal H3K9ac peaks, respectively. *p* values for motif enrichment in target sequences over background are mentioned. **d**–**f** NRF1 enrichment at NRF1 motifs in promoters of indicated insulin-upregulated genes and **g** a negative control region determined by ChIP-qPCR in MDA-MB-231 cells treated with insulin (100 nM) for 3 h or 6 h. IgG and FOXK2 antibody ChIPs serve as negative controls. Bars represent percent input pull-down in untreated (UT) and treated (3 h, 6 h) cells. Values are Mean + SEM from two independent experiments and three technical replicates from each experiment. Statistical significance was calculated using one-way ANOVA, Dunnett’s multiple comparisons test. ****p < 0.0001. **h**–**k** Quantification of expression of NRF1 (**h**) and NRF1 target genes (**i**–**k**) after siRNA-mediated NRF1 knockdown with or without insulin treatment for 6 h in MDA-MB-231 cells. Values are Mean + SEM from three independent experiments. Statistical significance was calculated using one-way ANOVA, Tukey’s multiple comparisons test. *p < 0.05, **p < 0.01, ****p < 0.0001, ns = nonsignificant

In order to further characterize the signal-dependent transcription factors involved in the altered gene expression network as well as chromatin acetylation induced by insulin, we performed transcription factor-binding motif enrichment analyses. Genes upregulated by insulin showed a significant enrichment of E-box elements that are bound by transcription factors such as MYC (c-Myc), CLOCK, USF1 and BHLHE40 (Fig. 5a). In addition to E-box elements, binding motifs for NRF1, ELF1, ELK1 and E2F transcription factors were enriched. Interestingly, MYC target genes were also enriched in the upregulated gene set (Fig. 4c), further suggesting the involvement of MYC in enhancing expression of genes in response to insulin. PI3K/AKT and MAPK pathways induced by insulin are known to enhance MYC activity by promoting the degradation of MAD1, an antagonist of MYC [37]. To test whether the transcription factors involved in insulin-induced gene expression were also involved in insulin-induced global histone acetylation changes, we performed similar motif enrichment analyses on H3K9ac peaks with significantly increased levels after insulin treatment. We categorized peaks with increased H3K9ac based on distance from TSS of known RefSeq genes. Proximal peaks were defined as peaks within 10 kb of a known TSS and distal peaks as those outside this window. Proximal peaks with increased H3K9ac signals showed enrichment of transcription factor-binding motifs for SP1, NRF1, ATF3, ELK1 and AP1 transcription factors among others (Fig. 5b). Distal peaks that exhibited increased H3K9ac signal in response to insulin showed enrichment of FOS, JUN and AP1 family transcription factor-binding sites (Fig. 5c). Interestingly, we found NRF1 binding sites to be enriched in upregulated gene promoters as well as proximal peaks with increased H3K9ac. NRF1 (nuclear respiratory factor 1) is required for expression of key metabolic genes regulating cellular growth including several nuclear-encoded mitochondrial genes [38]. Given the role of NRF1 in regulating metabolic genes, we tested whether NRF1 binding at its target sites was enhanced after insulin treatment. ChIP experiments showed that NRF1 binding indeed increased after 3 h insulin treatment at promoters of upregulated genes (Fig. 5d–f; Additional file 1: Figure S5). Increased NRF1 binding was also observed 6 h post-insulin treatment, however, to a lesser degree than at 3 h (Fig. 5d–f; Additional file 1: Figure S5) indicating an early response to insulin. Negative control ChIPs using rabbit IgG and antibody targeting FOXK2 did not exhibit the same insulin-induced binding as NRF1 at these genes (Fig. 5d–f). Furthermore, we evaluated the specificity of the NRF1 ChIP results by assessing binding at a control region that does not contain NRF1 binding motifs (Fig. 5g). These results indicate that NRF1 binding at promoters of NRF1 target genes could lead to increased histone acetylation at these regions. We further investigated whether NRF1 binding at these gene promoters was necessary for insulin-induced expression. We used siRNA against NRF1 and achieved a knockdown of expression by about 60% (Fig. 5h). RT-qPCR analyses of genes with promoter-bound NRF1 showed that NRF1 knockdown attenuated the insulin-induced expression of these genes (*CCDC86, LYAR* and *MYBBP1A*; Fig. 5i–k). These results indicate that NRF1 may regulate the metabolic capacity of cancer cells by integrating metabolic inputs from the environment to increase histone acetylation on chromatin that allow continuous transcription from these genes.

### Insulin increases mitochondrial biogenesis and ATP production

**Fig. 6:**
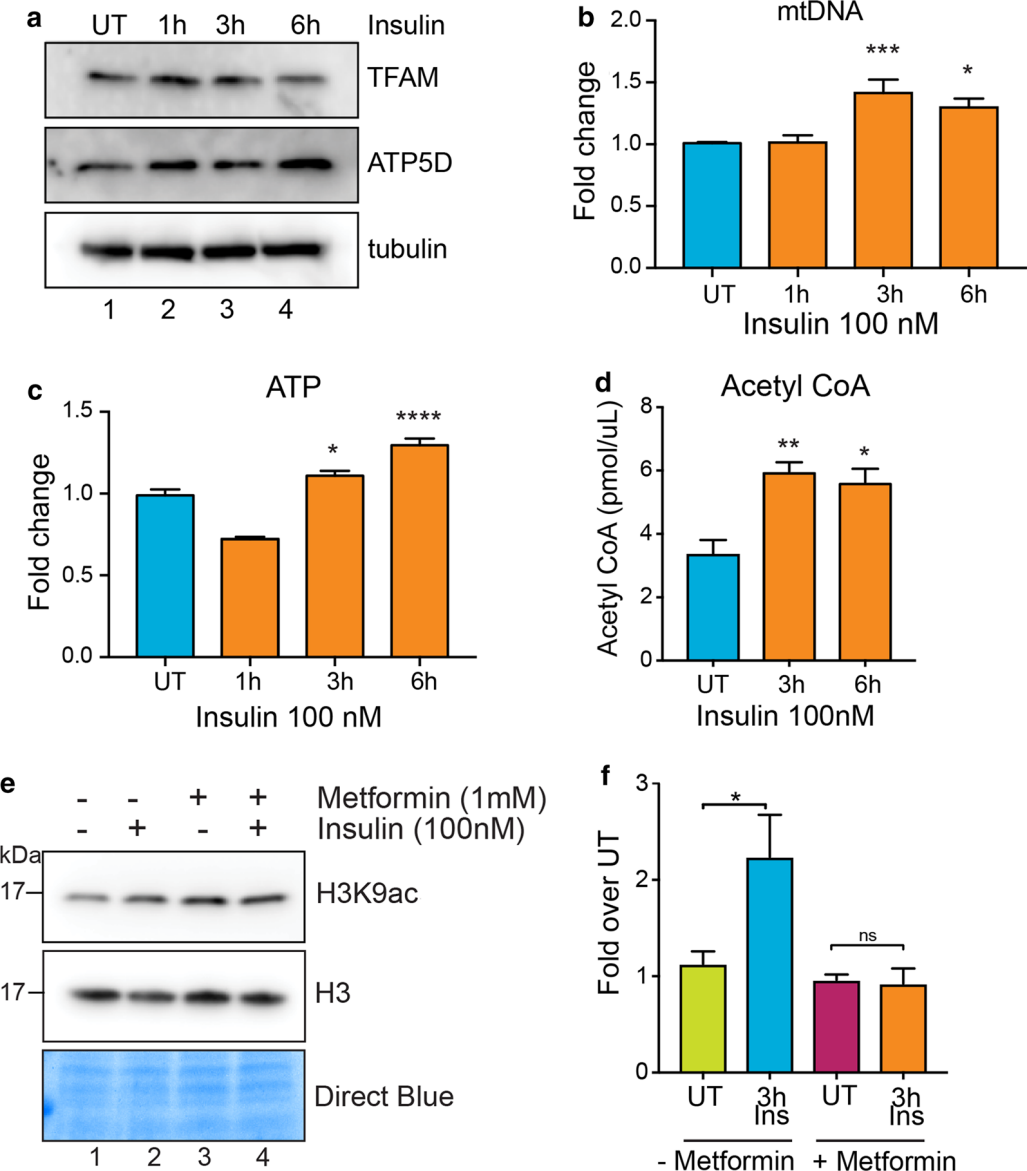
Insulin increases mitochondrial biogenesis and ATP production. **a** Western blot analysis using the indicated antibodies in MDA-MB-231 cell lysates treated with insulin (100 nM) for 1 h, 3 h or 6 h. **b** Bars show fold change in mitochondrial DNA content and **c** ATP levels in MDA-MB-231 cells treated with insulin (100 nM) for 1 h, 3 h or 6 h. **d** Acetyl-CoA levels in MDA-MB-231 cells treated with insulin (100 nM) for 3 h or 6 h. **e** Western blot analysis using H3K9ac antibody in MDA-MB-231 cells pretreated (lanes 3 and 4) or not (lanes 1 and 2) with 1 mM metformin (24 h) followed by insulin treatment for 6 h. **f** Densitometric quantification of H3K9ac/H3 western blot signals in (**e**). Data are represented as fold change to respective UT. **b**–**d**, **f** Values are Mean + SEM from three independent experiments. Statistical significance was calculated using one-way ANOVA, **b**–**d** Dunnett’s multiple comparisons test and **f** Tukey’s multiple comparisons test. *p < 0.05, **p < 0.01, ***p < 0.001, ****p < 0.0001, ns = nonsignificant

mTOR complex 1 (mTORC1) enhances mitochondrial biogenesis and activity by promoting translation of nuclear-encoded mitochondrial mRNAs including the components of Complex V and TFAM (transcription factor A, mitochondrial) [18]. To test whether insulin-induced histone acetylation increase was correlated with enhanced mitochondrial activity, we performed western blot analyses for TFAM and ATP5D (a subunit of the ATP synthase complex/Complex V) after insulin treatment. TFAM and ATP5D protein levels increase after 1 h insulin treatment (Fig. 6a). We further tested indicators of mitochondrial biogenesis and activity after insulin treatment. Mitochondrial DNA content is an indicator of mitochondrial number, and ATP levels are a measure of mitochondrial activity in cells [18]. Insulin increased the mitochondrial DNA content (Fig. 6b) as well as ATP levels (Fig. 6c) in MDA-MB-231 cells, indicating an enhancement in mitochondrial biogenesis and activity. In addition, we performed GC–MS to measure TCA cycle metabolites produced in the mitochondria. We observed increased levels of lactate and TCA cycle intermediates succinate, pyruvate, alpha-ketoglutarate, malate and citrate after 6 h of insulin treatment (Additional file 1: Figure S6). As acetyl-CoA is one of the key metabolites produced from citrate, we measured the levels of acetyl-CoA upon insulin stimulation. We find increased levels of acetyl-CoA after insulin treatment (Fig. 6d), indicating that acetyl-CoA levels might influence the levels of histone acetylation in the nucleus. To confirm that the observed increase in histone acetylation is caused by increased abundance of acetyl-CoA and not a global decrease in histone deacetylase (HDAC) activity, we assayed HDAC activity levels from nuclear extracts after insulin stimulation. We did not observe any significant change in HDAC activity caused by insulin (Additional file 1: Figure S7). These results confirm that insulin signaling-mediated increase in mitochondrial activity leads to increased acetyl-CoA levels and thereby enhances global histone acetylation levels in these cells.

To investigate whether insulin-induced chromatin changes occur through mitochondrial activity, we treated cells with metformin, which inhibits oxidative phosphorylation by inhibiting the mitochondrial Complex I activity [39]. Pre-treatment of cells with metformin prevented insulin-induced increases in H3K9 acetylation (Fig. 6e), indicating the potential significance of using metformin in triple-negative breast cancer patients with hyperinsulinemia to prevent insulin-mediated chromatin changes. These results indicate that mitochondrial activation is essential for histone acetylation induced by insulin.

### Insulin-induced reactive oxygen species (ROS) causes genome instability

**Fig. 7:**
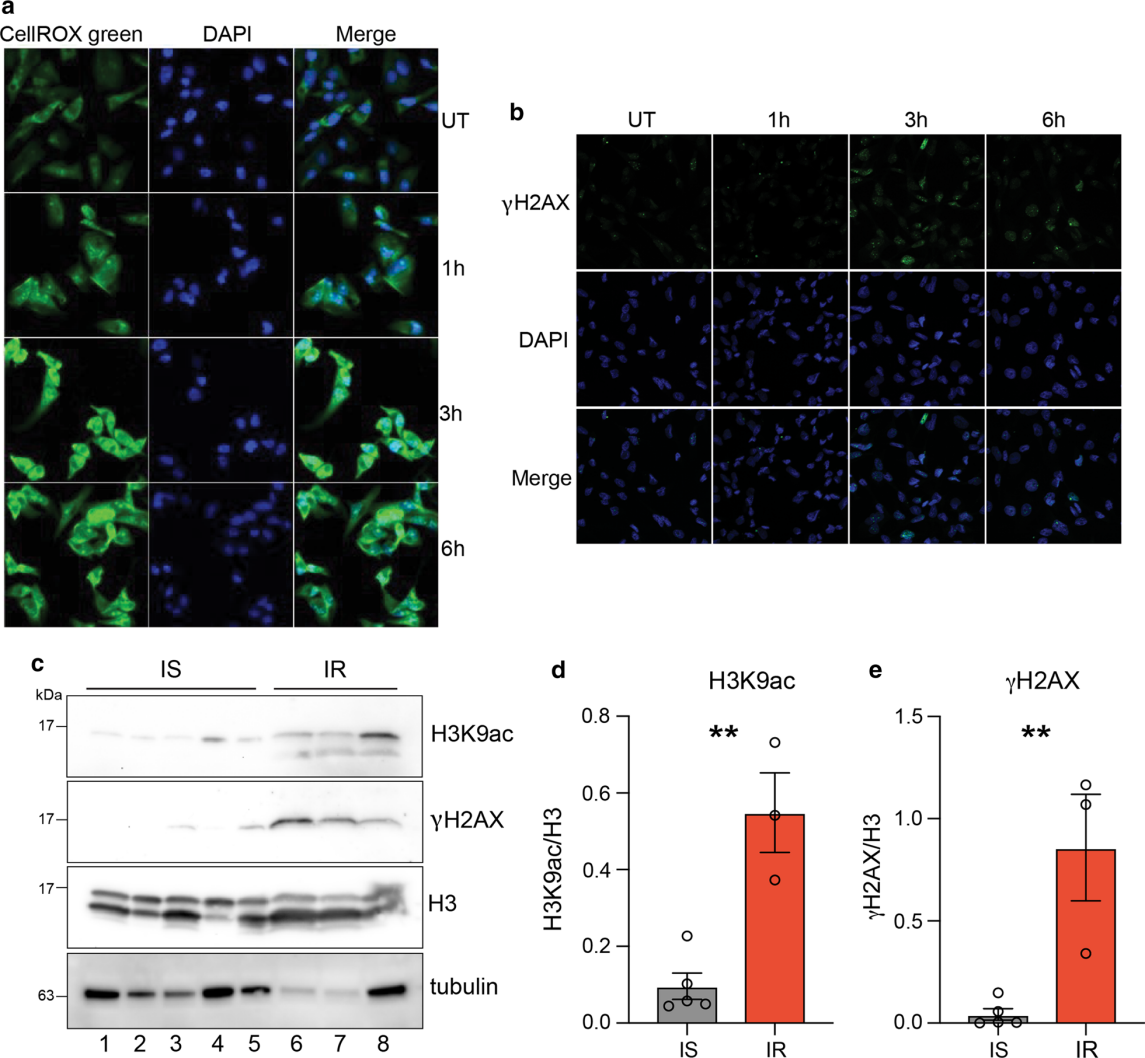
Insulin-induced reactive oxygen species (ROS) causes genome instability. **a** ROS induction in insulin-treated cells as measured by fluorescence signal from the CellROX green reporter. Nuclei are stained with DAPI. Magnification is ×40. **b** Representative immunofluorescence images showing intensity of γ-H2AX (DNA damage marker) (green) on MDA-MB-231 cells treated or untreated with Insulin (100 nM). Nuclei are stained with DAPI. Magnification is ×20. UT: untreated. (**c**) Western blot analysis for H3K9ac and γ-H2AX (DNA damage marker) on extracts from PBMCs isolated from insulin-sensitive (IS) and insulin-resistant (IR) individuals. **d** Densitometric quantification of H3K9ac/H3 western blot signals in (**c**). **e** Densitometric quantification of γH2AX/H3 western blot signals in (**c**). **d**–**e** Values are Mean + SEM; *n* = 5 for IS and *n* = 3 for IR PBMC lysates. Statistical significance was calculated using unpaired Student’s *t* test. **p < 0.01

Given that the mTOR pathway induces mitochondrial biogenesis and activity that leads to increased ROS production through the electron transport chain, we tested whether insulin induces ROS production in MDA-MB-231 cells. We measured ROS levels after insulin treatment using a fluorescent dye, CellROX green. Results showed that ROS levels significantly increased after 3 h of insulin treatment and remained high at 6 h (Fig. 7a). An increase in ROS production could be deleterious to cells, as the free radicals can cause DNA damage and mutation. We measured DNA damage using the DNA damage marker γ-H2AX in cells treated with insulin using immunofluorescence assays. We observed that the number of cells with γ-H2AX foci and the number of γ-H2AX foci per cell increased after 3 h insulin treatment (Fig. 7b). Interestingly, the number of cells with γ-H2AX foci decreased after 6 h indicating possible activation of repair pathways (Fig. 7b) as MDA-MB-231 cells harbor wild-type *BRCA1*. To investigate whether hyperinsulinemia is associated with increased histone acetylation and DNA damage in human samples, we measured the levels of H3K9ac and γ-H2AX in peripheral blood mononuclear cells (PBMCs) from an insulin-resistant and a healthy individual. We observed increased levels of H3K9ac and γ-H2AX in the insulin-resistant individual as compared to the insulin-sensitive individual (Fig. 7c, d and e) corroborating our in vitro results. Overall these data suggest that hyperinsulinemia might sensitize cells to DNA damage through increased ROS production and increased chromatin accessibility, thereby potentially promoting deleterious mutations in pre-neoplastic lesions.

## Discussion

Metabolic syndrome and its associated disorders are increasingly being recognized as enhanced risk factors for several types of cancers, including breast cancer. We investigated the impact of hyperinsulinemia, an important feature of metabolic syndrome, on chromatin and gene expression changes in TNBC cells. We observed an increase in chromatin-associated histone acetylation levels that was dependent on insulin-mediated signaling through the PI3K/AKT/mTOR pathway. We used a quantitative method of ChIP-seq (ChIP-Rx) to identify the regions associated with changes in H3K9ac in response to insulin and found genome-wide increases in H3K9ac occupancy at gene promoters, especially at genes that increased expression after insulin treatment. However, insulin-induced increase in histone acetylation at gene promoters was not always associated with an increase in gene expression, indicating that increased acetylation is not sufficient for increased transcription. Our observation is supported by a recent study investigating histone acetylation levels in response to high-glucose levels [40]. Interestingly, it has been proposed that histone acetylation may function as a capacitor for acetate/acetyl-CoA which could be utilized as an energy source or to balance the intracellular pH based on cellular condition [41].

Insulin has long been proposed to be involved in breast tumor progression. Fifty percent of breast tumors and most established breast cancer cell lines including TNBC cell lines overexpress IR [12, 42–45]. Insulin itself can induce proliferation in several breast cancer cell lines [46]. Our data further reinforce these observations. We find that insulin induces genes involved in ribosome biogenesis, transcription, splicing and metabolism that are regulated by MYC (c-Myc). Moreover, genes involved in apoptosis are downregulated in response to insulin.

Insulin-induced genes that showed increased H3K9ac signals were enriched for NRF1-binding sites. Indeed, NRF1 binding to these genes was induced by insulin and knockdown of NRF1 attenuated the insulin-induced expression of these genes. These results indicate that NRF1 binding is stimulated by insulin. NRF1 has been shown to exist as a phosphoprotein in cells. Phosphorylation of NRF1 by casein kinase II in vitro leads to increased DNA binding ability [47]. Presumably, insulin signaling through the PI3K/AKT/mTOR pathway induces NRF1 phosphorylation and thereby promotes binding to target sites.

Insulin enhances the utilization of glucose by inducing mitochondrial activity and biogenesis through mTOR. Tumor cells are known to reprogram metabolic pathways to support the increased demand for macromolecules required for uncontrolled proliferation in response to growth factors. Acetyl-CoA is a central metabolite that links glucose metabolism to lipid synthesis as well as regulation of chromatin [48]. Histone acetylation levels have been previously shown to correlate with acetyl-CoA abundance [49]. In proliferating cells, acetyl-CoA can be generated by a) oxidative decarboxylation of pyruvate from glycolysis; b) ATP-citrate lyase (ACLY) utilizing cytosolic citrate; and c) ACSS2 using acetate [48]. ACLY activity is enhanced by AKT-mediated phosphorylation, thereby establishing a link between insulin signaling, acetyl-CoA levels and histone acetylation in the nucleus [50]. Indeed, we observe increased acetyl-CoA abundance after insulin stimulation, indicating that the observed genome-wide increase in histone acetylation in response to insulin might be a result of increased acetyl-CoA abundance due to increased ACLY activity. Similar studies in pancreatic ductal carcinoma support a role for altered acetyl-CoA abundance through AKT-ACLY signaling by growth factors including insulin in promoting tumorigenesis [51].

Our findings also suggest that insulin induces ROS. This could be a result of increased mitochondrial activity or due to a decrease in scavenger proteins such as SOD2 (superoxide dismutase 2). Indeed, we observe a higher mitochondrial activity as well as decreased expression of SOD2 in response to insulin (RNA-seq results). Moreover, we find increased accumulation of DNA damage foci when the ROS levels peak after insulin treatment. The role of ROS in inducing gemomic instability in cells and promoting cancer cell survival as well as tumorigenesisis is well documented [52–54]. These results indicate that increased insulin signaling might predispose cells to deleterious mutations if they fail to repair the damage. This might be especially relevant in the context of BRCA1-mutated triple-negative breast cancer cells. We also observe elevated levels of DNA damage foci in individuals with elevated HbA1c levels. In support of this, a recent report suggests that individuals with increased HbA1c levels exhibit increased levels of DNA damage foci, genomic instability and KRAS mutations in the pancreas [55].

Given the link between hyperinsulinemia and the poor prognosis of breast cancer patients diagnosed with TNBC, understanding the cellular changes in response to hyperinsulinemia is important. Our finding that insulin drives hyperacetylation of histones in chromatin—thus impacting the transcriptome—highlights the impact insulin has within the nucleus.

## Conclusions

Our study offers novel insights into the connection between metabolism, signaling and nuclear processes in the context of insulin signaling. We show that mTOR signaling activated by insulin enhances mitochondrial activity. Acetyl-CoA and ROS metabolites produced as a result of mitochondrial activity impact nuclear processes such as chromatin accessibility and DNA damage. Furthermore, downstream signal-dependent transcription factors activated by PI3K/AKT/mTOR signaling, such as MYC and NRF1, stimulate transcription of cell proliferation and metabolic genes allowing cancer cell growth. Endogenous hyperinsulinemia may enhance tumor growth through these mechanisms as well. These results are important in understanding the metabolic pathways and gene networks regulated by insulin in triple-negative breast cancer cells and will help in designing therapeutics targeting cancer cells.

## Materials and methods

### Cell culture, reagents and antibodies

Human cell lines MDA-MB-231 (Cat No. HTB-26), AU565 (Cat No. CRL-2351) and *Drosophila* S2 cells (Cat No. CRL-1963) were purchased from American Type Culture Collection (ATCC, Manassas, VA, USA). T47D and HCC1954 cell lines were kind gifts from Dr. Mark LaBarge (City of Hope, CA, USA). MDA-MB-231 cells were grown in Dulbecco’s modified Eagle’s medium (DMEM) with high glucose (25 mM) (Cat. No. 25-500; Genesee Scientific, San Diego, CA); and AU565, T47D and HCC1954 were grown in RPMI-1640 medium (Cat. No. 25-506H; Genesee Scientific) at 37 °C, 5% CO_2_ in a humidified chamber. S2 cells were cultured in Schneider’s *Drosophila* medium (Cat. No. 21720024; Thermo Fisher Scientific, Waltham, MA) at 24 °C in a humidified chamber without CO_2_. All media were supplemented with 10% heat-inactivated fetal bovine serum (v/v) (SH30910.03, Fisher) and 1X antibiotics containing penicillin and streptomycin (Cat. No. 25-512, Genesee). Prior to insulin treatment, cells were serum depleted in DMEM high-glucose (25 mM) medium containing 0.2% BSA (serum depletion medium) for 24 h and then stimulated with 100 nM insulin (Cat No. I9278; Sigma-Aldrich, St. Louis, MO, USA) or left untreated (UT) for indicated time periods. All cell lines were routinely tested for mycoplasma contamination and used for no more than 10 passages. Where indicated, cells were treated with 50 μM LY294002 (Cell Signaling Technology; Cat No. 9901S), 20 nM rapamycin (Cayman Chemical; Cat No. 13346), 1 mM metformin (Sigma-Aldrich; Cat No. D150959) or vehicle DMSO for 1 h prior to insulin treatment (100 nM, 3 h). Primary antibodies used in this study are rabbit polyclonal and mouse monoclonal antibodies against acetylated H3 (acH3) (Cat Nos. ab47915; Abcam, Cambridge, UK), H3K9ac (ab4441; Abcam), H3K14ac (C10010-1; EpiGentek, Farmingdale, NY), H3 (ab1791; Abcam), phospho-AKT (2965S; Cell Signaling Technology, Danvers, MA), AKT (2920S; Cell Signaling Technology), phospho-S6K (9206S; Cell Signaling Technology), S6K (9202S; Cell Signaling Technology), TFAM (7495S; Cell Signaling Technology), ATP5D (ab97491; Abcam), NRF1 (ab34682; Abcam), γH2AX (NB100-78356; Novus Biologicals, Littleton, CO), tubulin (2125S; Cell Signaling Technology) and actin (Sigma, A5441). Secondary antibodies used are HRP-conjugated anti-rabbit or anti-mouse secondary antibodies (Abcam, Cat. Nos. ab6721 and ab6789, respectively) and Alexa-488-conjugated anti-mouse antibody (Cat No. A-11029, Thermo Fisher Scientific).

### ChIP-Rx

ChIP-Rx was performed as described in [35] with minor modifications detailed further in Additional file 1: Supplementary Materials and Methods.

### ChIP-seq analyses

Sequencing reads from each library were aligned to a combined reference genome (human + *Drosophila*) using bowtie [56]. The combined reference genome was generated as described in [35]. Bowtie alignment was done against the combined genome using parameters: -m 1 -e 70 -k 1 -n 2 –best –chunkmbs 200. About 6% of reads aligned to the dm3 genome and ~ 94% aligned to the hg19 genome. The number of reads aligned to human and *Drosophila* genome is reported in Additional file 1: Table S1. We identified a union set of 40,222 and 5716 H3K9ac peaks in the human and *Drosophila* cells, respectively. We normalized peak scores for the 40,222 human (hg19) peaks using hg19 aligned read counts (read count normalization) (Additional file 1: Figure S2A). Moreover, we used *Drosophila* (dm3) aligned read counts for normalizing peak scores (spike-in normalization) (Additional file 1: Figure S2B). Quite significantly, we observed greater changes in H3K9ac levels on peaks in 3 h and 6 h insulin-treated cells after spike-in normalization as compared to read count normalization (Additional file 1: Figure S1C and D). The effect of spike-in normalization was also evident in aggregate profiles of H3K9ac ± 2 kb around annotated transcription start sites (TSSs) (Additional file 1: Figure S1E and F). Overall spike-in normalization led to better conformity between replicates and revealed global increase in histone acetylation that could be quantified.

### Statistical analyses

Data are represented as mean and standard error of mean (Mean + SEM). Statistical analyses were performed using GraphPad Prism 7.0 software (GraphPad Prism Software Inc., San Diego, CA) and R. Normal distribution was confirmed using Shapiro–Wilk normality test before performing statistical analyses. For normally distributed data, comparison between two means was assessed by unpaired two-tailed Student’s *t* test and that between three or more groups were evaluated using one-way analysis of variance (ANOVA) followed by Tukey’s post hoc test. In the case of Student’s *t* test, *F* test was performed to check whether the variance in the groups compared was significantly different. For data where significantly different variances were observed, a *t* test with Welch correction was performed. For data that did not follow a normal distribution, Mann–Whitney test was performed for comparison between two groups and Kruskal–Wallis test followed by Dunn’s multiple comparisons test was performed for comparing more than two groups. A *p* value of < 0.05 was considered statistically significant. Figures were generated using Adobe Illustrator software (San Jose, CA, USA).

Other methods are described in Additional file 1: Supporting materials and methods.

## Additional file

**Additional file 1.** Supplemental information Supporting materials and methods section, including supplementary Figures S1–S8 and Tables S1–S2.

## Data Availability

The datasets generated in this study are available in the NCBI Gene Expression Omnibus (GEO) under accession number GSE124127.

## Abbreviations

TNBC: triple-negative breast cancer
PI3K: phosphoinositide 3-kinase
AKT: protein kinase B
mTOR: mechanistic target of rapamycin
T2D: type 2 diabetes
IR: insulin receptor
IGF-I: insulin-like growth factor 1
IGFBP1: insulin-like growth factor-binding protein 1
IGF-IR: insulin-like growth factor 1 receptor
TCA: tricarboxylic acid cycle
ROS: reactive oxygen species
S6K: ribosomal protein S6 kinase beta-1
ATP: adenosine triphosphate
mTORC1: mammalian target of rapamycin complex 1
TFAM: transcription factor A, mitochondrial
ChIP: chromatin immunoprecipitation
TSS: transcription start site
FOXO: forkhead box O
