# Supplementary material for: Hyperinsulinemia promotes aberrant histone acetylation in triple-negative breast cancer
Source: Epigenetics Chromatin. 2019 Jul 17;12:44. doi: 10.1186/s13072-019-0290-9 (PMC6636093; doi:10.1186/s13072-019-0290-9)
Supplement: Supplementary file 1 — Additional file 1. Supplemental information Supporting materials and methods section, including supplementary Figures S1–S8 and Tables S1–S2. [file 13072_2019_290_MOESM1_ESM.docx]

**­Hyperinsulinemia promotes aberrant histone acetylation in triple negative breast cancer**

Parijat Senapati^1^, Hiroyuki Kato^1^, Michael Lee^1,2^, Amy Leung^1^, Christine Thai^3^, Angelica Sanchez^3^, Emily J Gallagher^4^, Derek LeRoith^4^, Victoria L. Seewaldt^2,3^, David K. Ann^1,2^, Dustin E. Schones^1,2,*^

Authors’ email addresses in the order as given in author list: pasenapati@coh.org, hkato@coh.org, michlee@coh.org, aleung@coh.org, chthai@coh.org, angsanchez@coh.org, Emily.Gallagher@mssm.edu, derek.leroith@mssm.edu, vseewaldt@coh.org, dann@coh.org, dschones@coh.org

^1^Department of Diabetes Complications and Metabolism, Beckman Research Institute, City of Hope, Duarte, CA, 91010, USA

^2^Irell & Manella Graduate School of Biological Sciences, City of Hope, Duarte, CA, USA

^3^Department of Population Sciences and Beckman Institute, City of Hope, Duarte, CA, 91010, USA

^4^Division of Endocrinology, Diabetes and Bone Diseases, Icahn School of Medicine at Mount Sinai, New York, NY, 10029, USA

*Corresponding Author:

Dustin E. Schones (dschones@coh.org)

Dustin Schones, Ph.D.

Department of Diabetes Complications and Metabolism

City of Hope

Duarte, CA, 91010

Email: dschones@coh.org

Phone: 626-218-1319

Fax: 626-358-7703

**Supplementary Materials and Methods**

**Western Blot**

Cells were seeded at a density of 0.5 ×10^6^ cells per well in six well plates. The medium was changed to serum depletion medium 48h post-seeding. Insulin (100nM) treatment was done after 24h of serum depletion for indicated time durations. Cells were collected by scraping and centrifugation at 800×g for 5 mins at 4°C. Cell pellet was washed once with PBS and resuspended in 20 cell volumes of 1X SDS sample buffer. Cell lysates were prepared by performing two iterations of vortexing and heating at 95°C for 5 mins. Lastly, the cell lysates were sonicated using a Bioruptor^R^ pico (Diagenode, Leige, Belgium) for three cycles (30 sec on/30 sec off) at room temperature (RT) and cleared by centrifugation at 16000×g for 5 mins at RT. Western blotting was performed by running cell lysates on a gradient SDS-PAGE gel and transferring onto a PVDF membrane. Membranes were stained with Direct Blue 71 dye (Sigma) as decribed previously (1). Western blots were probed with indicated antibodies overnight at 4°C. After primary antibody incubation, blots were incubated with HRP-conjugated anti-rabbit or anti-mouse secondary antibodies and protein bands were visualized using chemiluminescence detection (Cat. No. 34076, Thermofisher Scientific). Densitometric quantification of western blot signals was done using Image J software as described previously (2).

**Chromatin fractionation**

Chromatin fractionation was performed using the method described in (3). Briefly, cells were collected from six well plates after insulin treatment by scraping and centrifugation at 200×g for 2 mins. Cell pellet was washed twice with PBS and resuspended and incubated in 100 μl buffer A (10mM HEPES, pH 7.9, 10mM KCl, 1.5mM MgCl_2_, 0.34M sucrose, 10% glycerol, 1mM DTT) supplemented with protease inhibitors and 0.1% Triton X-100 for 8 mins on ice. Nuclei were isolated by centrifugation at 1300×g for 5 mins at 4°C. The nuclear pellet thus obtained was washed once with buffer A and then resuspended in 100 μl buffer B (3mM EDTA, 0.2mM EGTA,1mM DTT) supplemented with protease inhibitors and incubated for 30 mins on ice. The nuclear suspension was centrifuged at 1700×g for 5 mins at 4°C to obtain nuclear soluble fraction in the supernatant and chromatin fraction in pellet. The chromatin fraction was resuspended in 1X SDS sample buffer and boiled at 95°C for 5 mins.

**Mitochondrial DNA and ATP measurement**

For mitochondrial DNA quantification, genomic DNA and mitochondrial DNA was extracted from insulin treated cells using DNeasy Blood and Tissue kit (Qiagen, Hilden, Germany). Genomic and mitochondrial DNA were quantified by qPCR using mitochondrial and genomic DNA specific primers; namely, cytochrome B and RPL13A (Table S2).

For ATP measurement, MDA-MB-231 cells untreated or treated with insulin in 12 well plates were lysed by boiling in deionized water for 5 mins. The lysate was clarified by centrifugation at 16000×g for 10mins at 4°C. ATP levels were measured using the luciferase-based ATP determination kit (Cat No. A22066; Thermofisher Scientific).

For Acetyl-CoA measurement, MDA-MB-231 cells untreated or treated with insulin in 100 mm dishes were harvested by scraping and lysed in RIPA buffer. Cell lysates were deproteinized as per manufacturer’s instructions using a deproteinization kit (Cat No. K808; Biovision). Deproteinized samples were used to meaure acetyl CoA levels using a fluoremetric assay kit (Cat No. K317, Biovision) as per the manufacturer’s instructions. HDAC activity was measured from nuclear lysates using a fluoremetric assay kit (Cat No. K330, Biovision).

**ChIP-Rx**

ChIP-Rx was performed as described in (4) with minor modifications. MDA-MB-231 cells were seeded at a density of 1 ×10^6^ cells in 60mm dishes and treated with 100nM insulin as described above. After treatment, cells were cross-linked using 1% formaldehyde for 10 mins at RT, followed by addition of 0.125 M glycine for 5 mins to stop the reaction. In parallel, S2 cells were crosslinked at a density of 1 ×10^6^ cells per ml using 1% formaldehyde. Crosslinked cells were then washed twice with ice-cold PBS supplemented with protease inhibitors. MDA-MB-231 and S2 cells were resuspended in parallel in cold lysis buffer 1 (140mM NaCl, 1mM EDTA, 50mM HEPES pH 7.5, 10% Glycerol, 0.5% NP-40, 0.25% Triton-X-100) supplemented with protease inhibitors (COmplete, Cat. No.11873580001, Sigma) and incubated on ice for 10 mins. Next, centrifugation was performed at 800×g for 5 mins at 4°C to pellet nuclei. Nuclear pellets were then resuspended in parallel in lysis buffer 2 (10mM Tris pH 8.0, 200mM NaCl, 1mM EDTA, 0.5mM EGTA) supplemented with protease inhibitors and incubated for 10 mins on ice. At this step, *Drosophila* nuclear suspension was added to each untreated and treated human MDA-MB-231 nuclear suspension at a ratio of (1 *Drosophila* cell per 2 human cells) or 1.5×10^6^ S2 cells to 3×10^6^ MDA-MB-231 cells. The composite cell nuclei were then pelleted at 800×g for 5 mins at 4°C.

Composite cell pellets were resuspended in SDS lysis buffer (1% SDS, 10mM EDTA, 50mM Tris-HCl, pH 8) supplemented with protease inhibitors and subjected to sonication using a Bioruptor^R^ pico for six cycles (30 sec on/30 sec off) to produce DNA fragments of 200–500 bp in length. Sheared chromatin was clarified by centrifugation at 16000×g for 5mins at 4°C. 0.3 ×10^6^ cell equivalents were diluted with ten volumes of cold ChIP-dilution buffer (0.01% SDS, 1.1% Triton X-100, 1.2 mM EDTA, 16.7 mM Tris-HCl, pH 8, 167 mM NaCl) supplemented with protease inhibitors and used for each immunoprecipitation. About 10% of the chromatin from each ChIP reaction was saved as input. ChIP assays were performed with 5 μg anti-H3K9ac antibody and 25μl of magnetic protein G Dynabeads (Cat. No. 10004D; Thermofisher Scientific) which were incubated overnight at 4°C. 5 μg rabbit IgG was used for control ChIPs. Magnetic beads were washed successively with low salt buffer (0.1% SDS, 1% Triton X-100, 2mM EDTA, 20mM Tris-HCl, pH 8, 150 mM NaCl), high salt buffer (0.1% SDS, 1% Triton X-100, 2mM EDTA, 20mM Tris-HCl pH 8, 500mM NaCl), LiCl buffer (250 mM LiCl, 1% NP40, 1% NaDOC, 1mM EDTA, 10mM Tris-HCl, pH 8), and twice with TE buffer (10mM Tris-HCl pH 8, 1mM EDTA). Elution buffer (1% SDS and 100mM NaHCO_3_) was added to the washed beads, and the bead solution was incubated at RT for 30 mins. In parallel, the saved input was also diluted in Elution buffer. The DNA-protein complexes were then reverse cross-linked by adding 200mM NaCl, 20 µg Proteinase K (Cat. No. P4850; Sigma-Aldrich) and incubating at 65ºC for 4 hours. Subsequently, 20 µg of RNase A (Cat No. EN0531; ThermoFisher Scientific) was added and further incubated for 15 mins at 37ºC. The immunoprecipitated DNA was extracted using phenol-chloroform and ethanol precipitation. Resultant ChIP DNA was quantified using Quant-iT™ dsDNA Assay Kit (Cat No. Q33120; ThermoFisher Scientific) and used for library preparation.

ChIP-seq libraries were made using Illumina Tru-Seq library preparation kit (Illumina, San Diego, CA) and multiplexing barcodes compatible with Illumina HiSeq 2500 technology. About 50 million single-end reads of length 51 bp were generated from each ChIP-seq library.

For ChIP-qPCR analyes, ChIPs were performed as described above using 0.5 ×10^6^ cells and 5 μg of NRF1 (ab34682; Abcam) or FOXK2 (ab5298) antibodies followed by real time PCR analyses using specific primers listed in Table S2.

**RNA-seq analyses**

Total RNA was isolated from insulin treated cells or from tumors tissues using NucleoSpin® RNA kit (Macherey-Nagel, Germany) with on-column DNase I digestion. PolyA-enriched RNA was isolated and used for library preparation using TruSeq RNA library Prep kit (Illumina). About 50 million single-end reads of length 51 bp were generated for the MDA-MB-231 samples. Raw sequences were aligned to the hg19 reference genome using HISAT2 2.1.0 (5) using default parameters. Stringtie 1.3.4 (6) was used with default parameters to assemble transcripts using the Genocde v19 transcript annotation. Assembled transcripts from all libraries were further merged using --merge option in Stringtie. Merged transcript abundances were measured using bedtools coverage and DESeq2 package (7) was used to normalize counts and identify differentially expressed genes (log2 fold change ≥ 0.5 and padj < 0.1). Gene Set Enrichment Analysis (GSEA) (8) was used to determine significantly altered gene ontology and pathways. For validation of RNA-seq data, 1μg total RNA was used to synthesize cDNA using High-Capacity cDNA Reverse Transcription Kit (Cat No. 4368814; ThermoFisher Scientific). Gene expression was analyzed by quantitative PCR (qPCR) using KAPA SYBR® Fast ROX Low qPCR Master (Cat No. KM4117, Kapa Biosystems, Inc., Wilmington, MA) using gene-specific primers (Table S2). Relative gene expression between groups was determined using 2^^-ΔΔCt^ method after normalization with *PPIA* levels.

For siRNA-mediated NRF1 knockdown studies, 30 nM siRNA against NRF1 (ON-TARGETplus NRF1 siRNA; Cat No. L-017924-00-0005; Dharmacon) was transfected into cells seeded in 12 well plate using Lipofectamine RNAiMAX Transfection Reagent (Cat. No. 13778150; Thermofisher Scientific) as per manufacturer’s instructions. Medium was changed to serum depletion medium 48h post-transfection and insulin (100 nM) treatment was performed for 6h. Total RNA was extracted and mRNA expression changes were quantified as described above using specific primers listed in Table S2.

**Visualization of ChIP-seq data and additional analyses**

Reads aligning to the hg19 genome were filtered out from the aligned bam files and peaks were called for each library with respective Input using macs2 (9) with broad peak calling option and q < 0.1 for broad regions and q < 0.05 for narrow regions. Peaks were annotated using annotatePeaks.pl command in homer (10) and assigned to the nearest hg19 RefSeq gene TSS. Wiggle tracks were generated using custom scripts, normalized by the number of dm3 aligned reads and visualized on the UCSC Genome Browser. Heatmaps were generated using Java TreeView (11) and aggregate profiles were made using deepTools (12). Motif enrichment analysis was performed using findMotifsGenome.pl command in homer.

**Immunofluorescence analyses**

MDA-MB-231 cells were grown on cover slips coated with poly-L-lysine at 37°C in a 5% CO_2_ incubator. After treatment, the DMEM medium was aspirated out and the cell layer was washed twice with PBS. The cells were fixed with 4% para-formaldehyde in PBS for 10 mins at RT. The para-formaldehyde solution was removed and quenched with 100mM Tris pH 7.2 for 5 mins at RT. The fixed cells were subsequently permeabilized by incubating in 0.1% Triton X-100 solution in PBS for 10 mins. The cells were then washed twice with PBS at 5 mins intervals and blocked using the blocking solution (10% FBS, 3% BSA in PBS containing 0.1% Triton X-100), for 45 mins at 37°C. After incubation, the blocking solution was replaced with anti-γH2AX antibody (Novus biologicals, Cat No. NB100-78356, 1 in 1000 dilution) for 30 mins at 37°C. The cells were then washed with the wash buffer (PBS containing 0.1% Triton X-100) twice for 5 mins each. Subsequently the cells were incubated with Alexa-488 conjugated anti-mouse antibody (Cat No. A-11029, 1 in 1000 dilution; ThermoFisher Scientific) for 30 mins at 37°C. The cells were washed with the wash buffer twice for 5 mins each.

For reactive oxygen species detection, insulin treated, or untreated cells seeded on coverslips were treated with 5 μM CellROX Green reagent (Cat No. C10444; ThermoFisher Scientific) for 30 mins in culture medium at 37°C. Subsequently, the culture medium was aspirated, and cells were washed thrice with PBS. Nuclei were stained using 10 μg/ml DAPI solution (Cat No. D1306; ThermoFisher Scientific) for 5 mins in the dark, followed by two washes with PBS. The coverslips were then inverted onto a microscopic slide over a drop of 70% Glycerol (in PBS) for visualization. The Alexa, DAPI and CellROX Green fluorescence were visualized with a Carl Zeiss confocal laser scanning microscope LSM 710 META. Images were captured using Zen software. ImageJ software was used to process the images. The images for comparative studies were captured at identical microscope settings.

**Human samples**

Blood samples were obtained from patients after an 8h fasting period following institutional guidelines at the City of Hope (IRB no. 15418 and 18306) in purple-top EDTA vacutainer tubes after obtaining written informed consent. Insulin resistance was identified by measuring Hemoglobin A1C (HbA1c) using HPLC method (13). HbA1C levels between 5.7-6.3 were used to define insulin-resistance. All individuals were within ages of 18-55. PBMCs were isolated from whole blood using Ficoll-Paque method. Briefly, whole blood was diluted 1:1 with PBS containing 2% FBS, layered on top of 15 ml Ficoll in SepMate-50 tubes (Cat No. 15460; Stemcell Technologies) and spun down at 1200×g for 10 mins. The white buffy coat containing PBMCs were collected and washed twice with PBS containing 2% FBS and spun down at 1200×g for 10 mins to remove platelets. 0.5 ×10^6^ PBMCs were lysed in 20 cell volumes of 1X SDS sample buffer and processed for western blot analyses.

**Tumor xenograft studies**

Animal studies were performed at the Icahn School of Medicine at Mount Sinai (ISMMS) Center for Comparative Medicine and Surgery. All studies were approved by the ISMMS Institutional Animal Care and Use Committee. All animals used for the studies, were female, on an FVB/n background. The immunodeficient hyperinsulinemic mice were generated as previously described by crossing the recombination-activating gene 1 (*Rag1*) knockout mice with the muscle creatinine kinase promoter expressing dominant-negative *Igf1r* (MKR) mice (14). The metabolic phenotyping of the Rag1 knockout (Rag1^-/-^) / MKR mice and control Rag1^-/-^ mice has been characterized previously (14). Mice for this study were maintained on regular diet (PicoLab Rodent Diet 20, 5053), with free access to water and a 12-hour light / dark cycle. 5×10^6^ MDA-MB-231 tumor cells were injected into the inguinal mammary fat pad of *Rag1*^-/-^ and *Rag1*^-/-^/MKR female mice aged between 8 and 10 weeks. MDA-MB-231 tumor growth was measured as previously described (15). At the end of the study, tumors were dissected and flash frozen in liquid nitrogen for further analysis.

**References**

1. Hong HY, Yoo GS, Choi JK. Direct Blue 71 staining of proteins bound to blotting membranes. Electrophoresis. 2000;21(5):841-5.

2. Shandilya J, Senapati P, Hans F, Menoni H, Bouvet P, Dimitrov S, et al. Centromeric histone variant CENP-A represses acetylation-dependent chromatin transcription that is relieved by histone chaperone NPM1. J Biochem. 2014;156(4):221-7.

3. Mendez J, Stillman B. Chromatin association of human origin recognition complex, cdc6, and minichromosome maintenance proteins during the cell cycle: assembly of prereplication complexes in late mitosis. Mol Cell Biol. 2000;20(22):8602-12.

4. Orlando DA, Chen MW, Brown VE, Solanki S, Choi YJ, Olson ER, et al. Quantitative ChIP-Seq normalization reveals global modulation of the epigenome. Cell Rep. 2014;9(3):1163-70.

5. Kim D, Langmead B, Salzberg SL. HISAT: a fast spliced aligner with low memory requirements. Nat Methods. 2015;12(4):357-60.

6. Pertea M, Pertea GM, Antonescu CM, Chang TC, Mendell JT, Salzberg SL. StringTie enables improved reconstruction of a transcriptome from RNA-seq reads. Nat Biotechnol. 2015;33(3):290-5.

7. Love MI, Huber W, Anders S. Moderated estimation of fold change and dispersion for RNA-seq data with DESeq2. Genome Biol. 2014;15(12):550.

8. Subramanian A, Tamayo P, Mootha VK, Mukherjee S, Ebert BL, Gillette MA, et al. Gene set enrichment analysis: a knowledge-based approach for interpreting genome-wide expression profiles. Proc Natl Acad Sci U S A. 2005;102(43):15545-50.

9. Zhang Y, Liu T, Meyer CA, Eeckhoute J, Johnson DS, Bernstein BE, et al. Model-based analysis of ChIP-Seq (MACS). Genome Biol. 2008;9(9):R137.

10. Heinz S, Benner C, Spann N, Bertolino E, Lin YC, Laslo P, et al. Simple combinations of lineage-determining transcription factors prime cis-regulatory elements required for macrophage and B cell identities. Mol Cell. 2010;38(4):576-89.

11. Saldanha AJ. Java Treeview--extensible visualization of microarray data. Bioinformatics. 2004;20(17):3246-8.

12. Ramirez F, Ryan DP, Gruning B, Bhardwaj V, Kilpert F, Richter AS, et al. deepTools2: a next generation web server for deep-sequencing data analysis. Nucleic Acids Res. 2016;44(W1):W160-5.

13. Davis JE, McDonald JM, Jarett L. A high-performance liquid chromatography method for hemoglobin A1c. Diabetes. 1978;27(2):102-7.

14. Zelenko Z, Gallagher EJ, Antoniou IM, Sachdev D, Nayak A, Yee D, et al. EMT reversal in human cancer cells after IR knockdown in hyperinsulinemic mice. Endocr Relat Cancer. 2016;23(9):747-58.

15. Shlomai G, Zelenko Z, Antoniou IM, Stasinopoulos M, Tobin-Hess A, Vitek MP, et al. OP449 inhibits breast cancer growth without adverse metabolic effects. Endocr Relat Cancer. 2017;24(10):519-29.

**Supplementary figures**

**
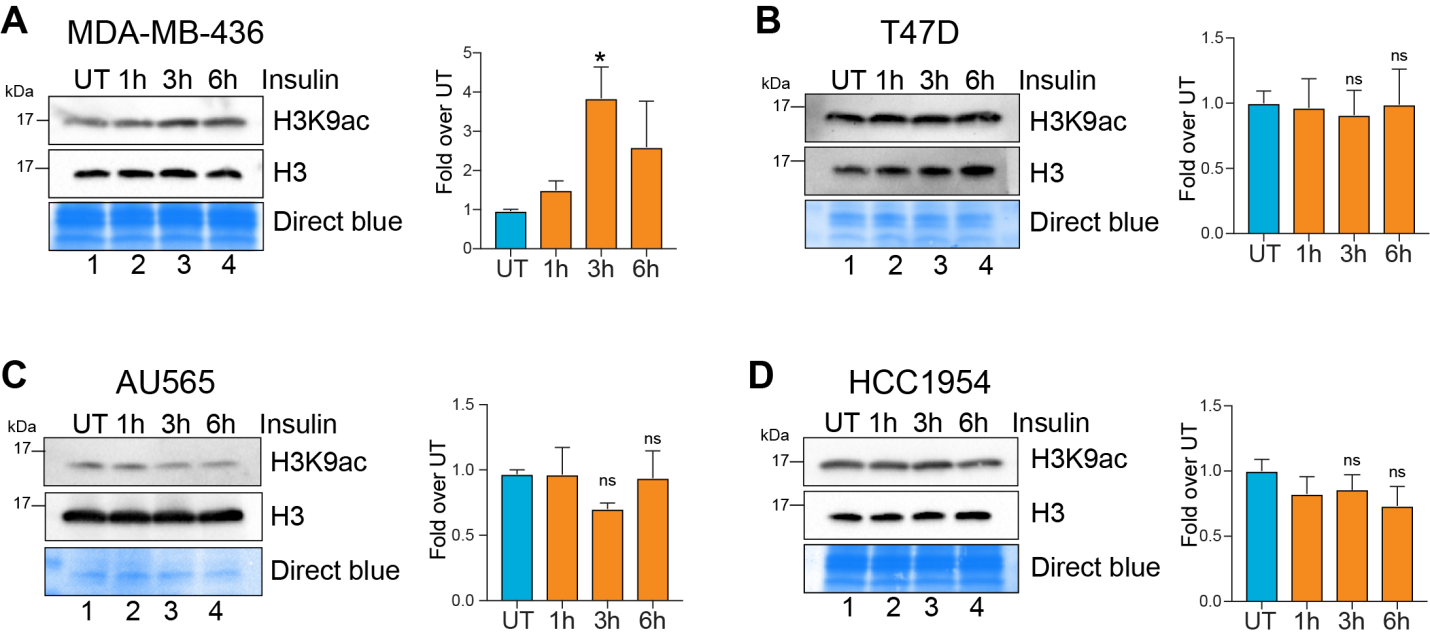
**

**Figure S1:** (A) Western blot analysis using the indicated antibodies in MDA-MB-436 (TNBC cell line) cell lysates treated with insulin (100 nM) for 1h, 3h or 6h. (B) Western blot analysis using the indicated antibodies in non-TNBC cell lines T47D, (C) AU565 and (D) HCC1954 treated with insulin (100 nM) for 1h, 3h or 6h. Densitometric quantification for H3K9ac/H3 signals is shown for each western blot on the right. Statistical significance was calculated using one-way ANOVA, Dunnett’s multiple comparisons test. *p<0.05, ns: non-significant.


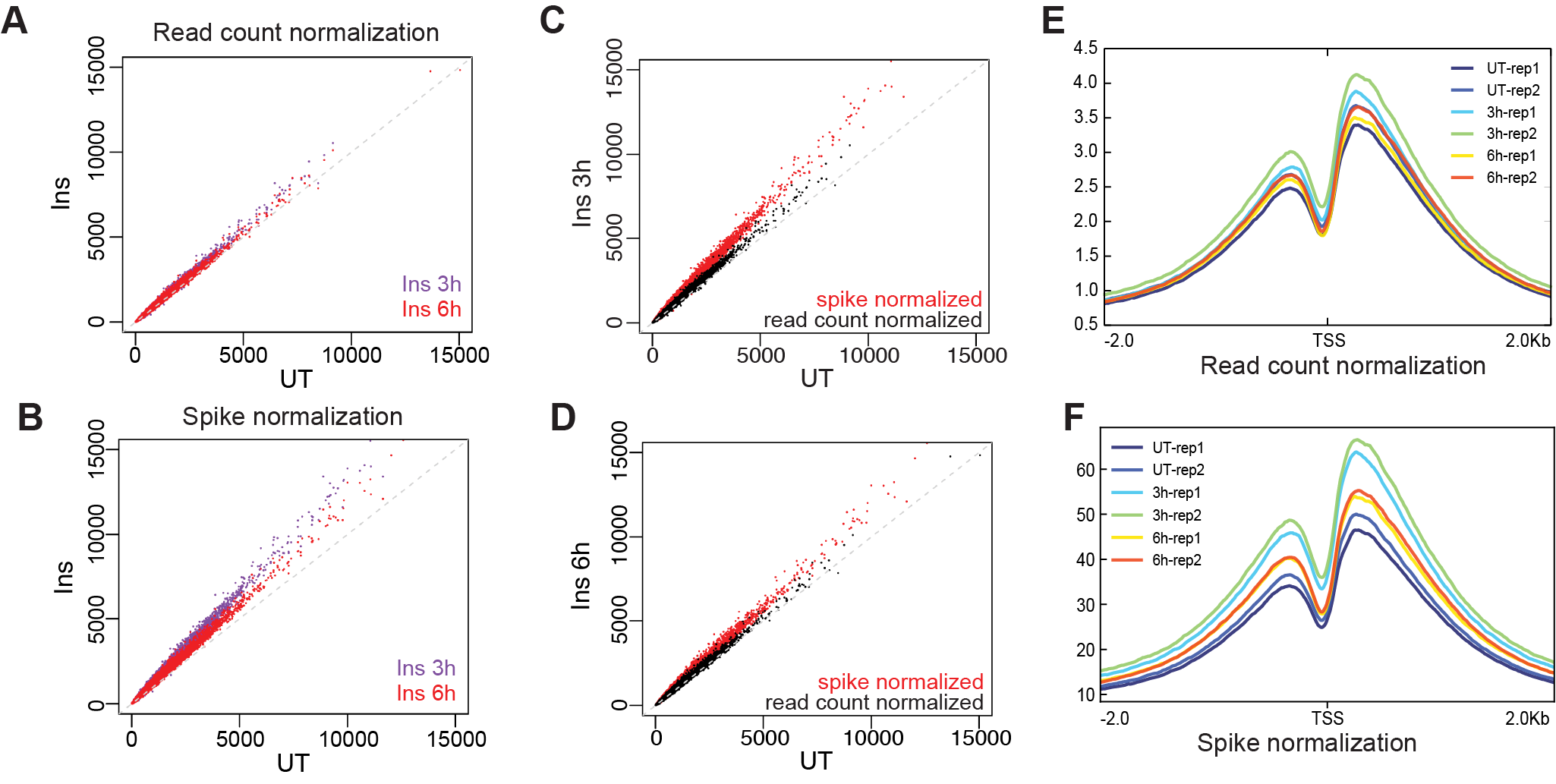


**Figure S2:** (A) Scatterplots showing the peak scores in UT versus 3h (purple) and 6h (red) insulin treatment normalized with total aligned read counts (hg19) and (B) spike-in normalization (aligned read counts from dm3 genome). (C) Scatterplots showing the peak scores in UT versus 3h and (D) UT vs 6h insulin treatment normalized with total aligned read counts (hg19) (black) or spike-in normalization (red). (E) Aggregate profile of H3K9ac signals around transcription start sites (TSS) normalized using read count normalization or (F) spike normalization in all ChIP-seq libraries.


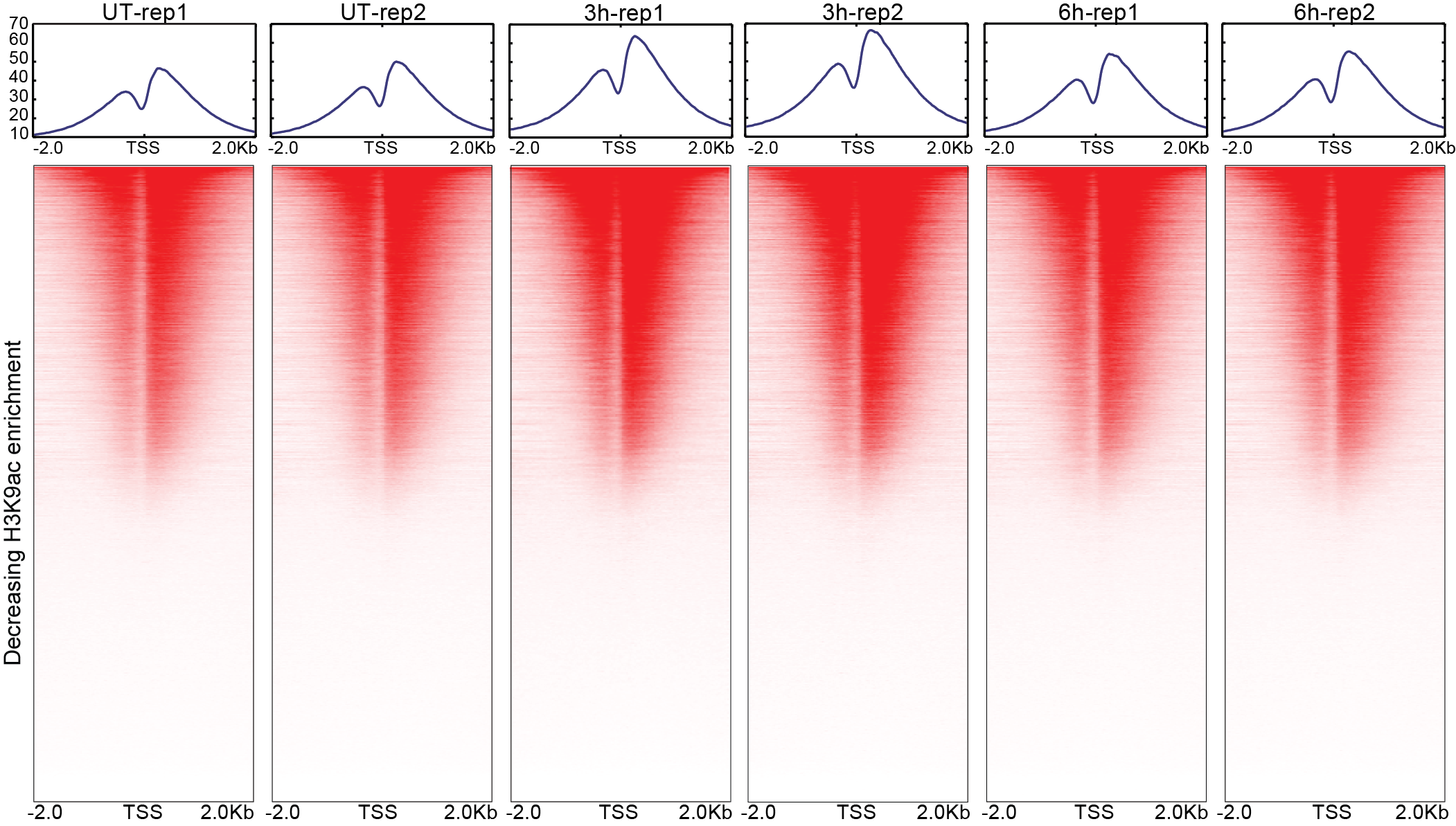


**Figure S3:** Heat maps and average profiles showing the distribution and enrichment of H3K9ac tags at promoter regions (±2kb from TSS of all transcripts in UT and 3h and 6h insulin treated samples.


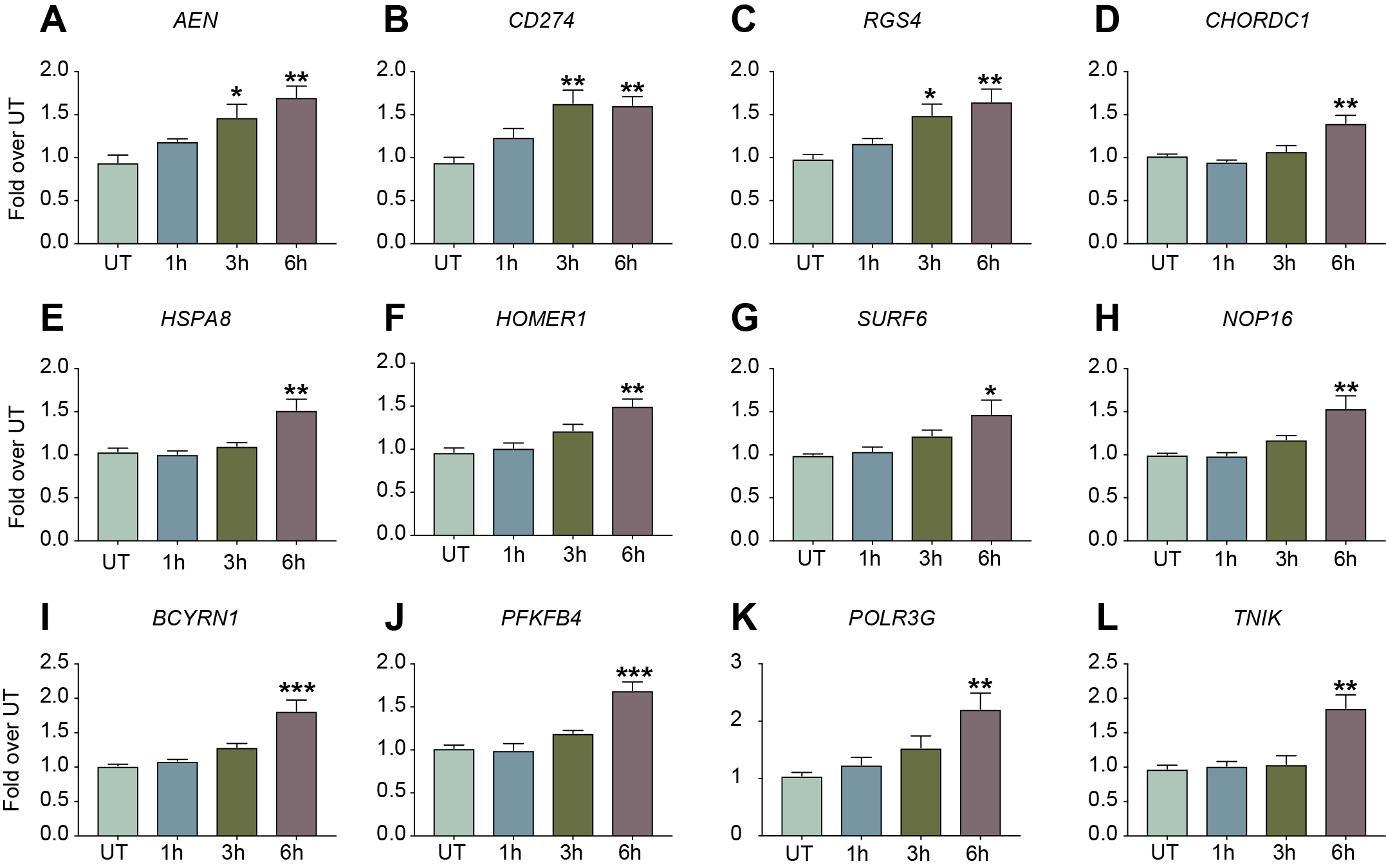


**Figure S4:** RT-qPCR quantification of mRNA expression for genes upregulated by insulin stimulation (100 nM) in MDA-MB-231 cells for 1h, 3h or 6h. UT: Untreated. Values are Mean+SEM from three independent experiments. Statistical significance was calculated using one-way ANOVA, Dunnett’s multiple comparisons test. *p<0.05, **p<0.01, ***p<0.001.


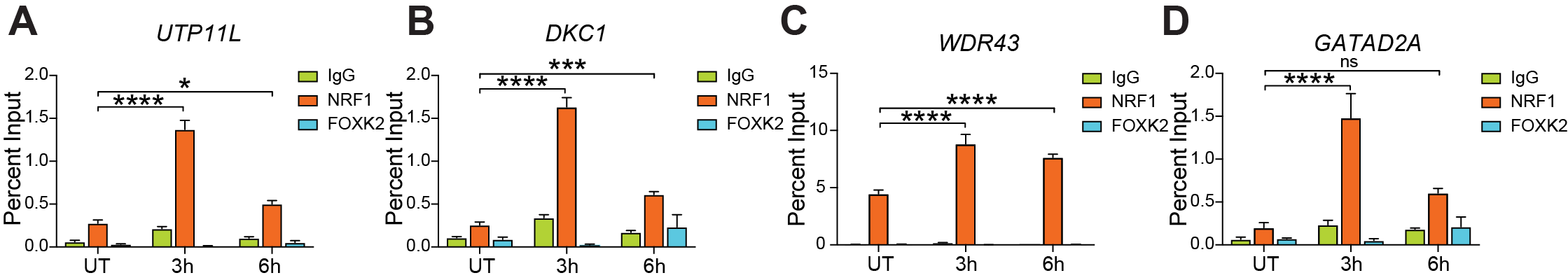


**Figure S5:** NRF1 enrichment at NRF1 motifs in promoters of indicated insulin-upregulated genes determined by ChIP-qPCR in MDA-MB-231 cells treated with insulin (100 nM) for 3h or 6h. IgG and FOXK2 antibody ChIPs serve as negative controls. Bars represent percent input pulldown in untreated (UT) and treated (3h, 6h) cells. Values are Mean+SEM from two independent experiments and three technical replicates from each experiment. Statistical significance was calculated using one-way ANOVA, Dunnett’s multiple comparisons test. *p<0.05, ***p<0.001, ****p<0.0001, ns=non-significant.


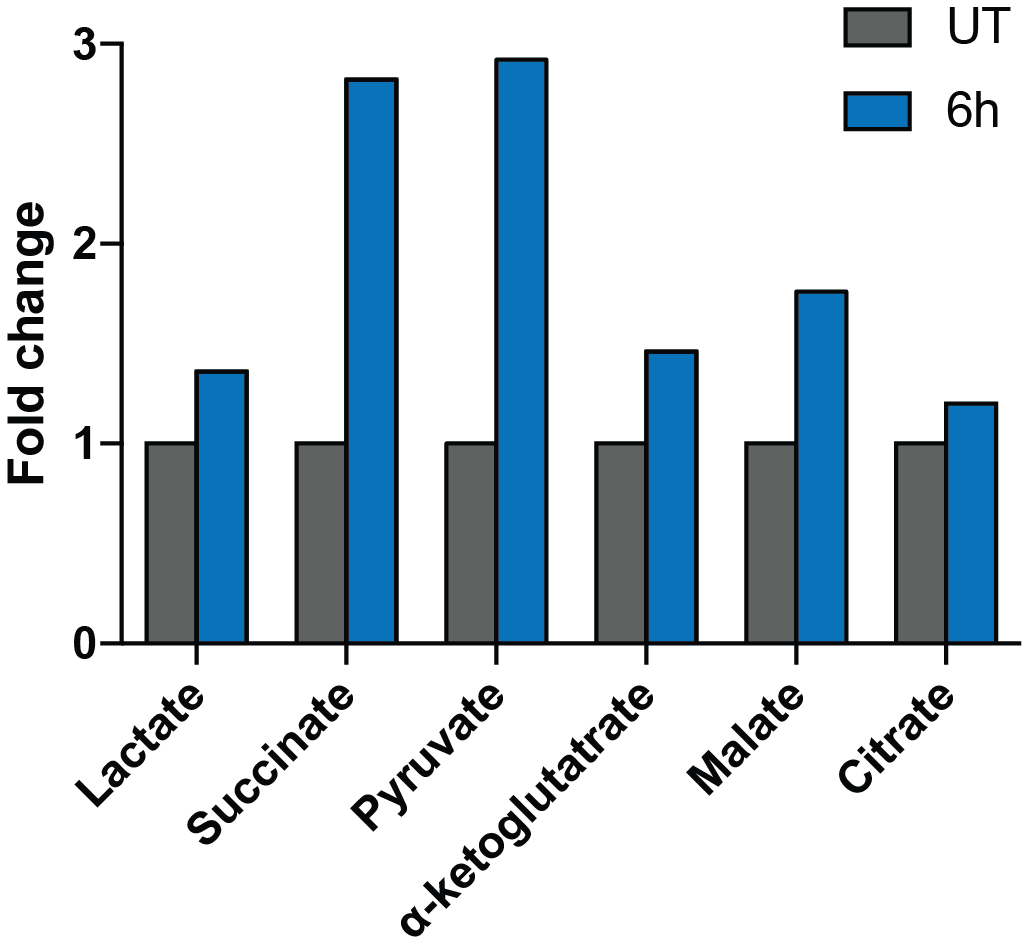


**Figure S6:** Bars represent fold change in levels of indicated metabolites in cells treated with 100nM insulin for 6h. UT: Untreated.


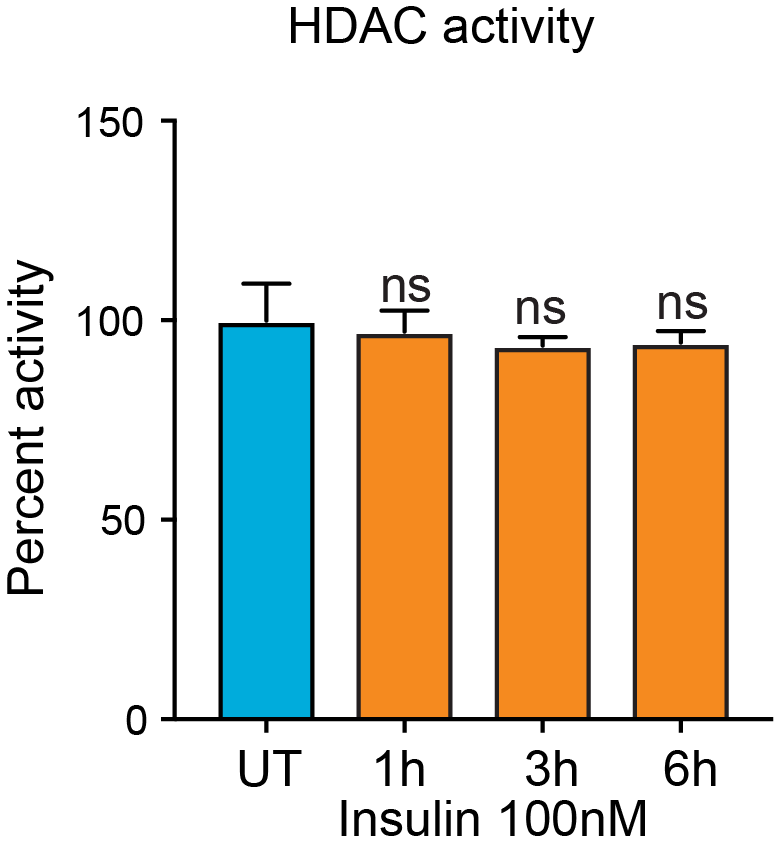


**Figure S7:** Bars represent percent HDAC activity in MDA-MB-231 cells treated with 100nM insulin for 1h, 3h or 6h. Values are Mean+SEM; n=3. Statistical significance was calculated using one-way ANOVA, Dunnett’s multiple comparisons test. UT: Untreated, ns: non-significant.


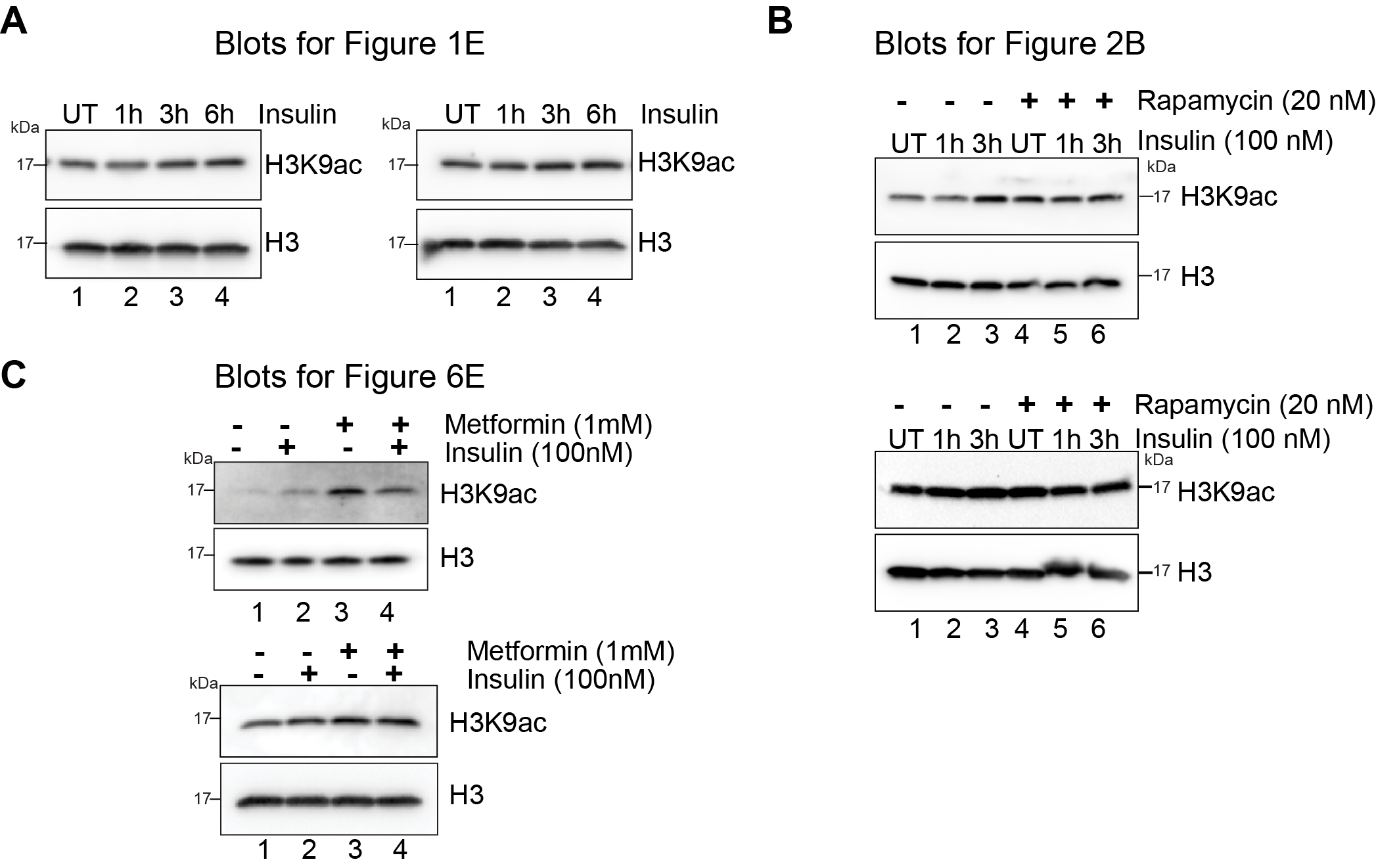


Figure S8: (A) Blots used for quantification shown in Figure 1F (B). Blots used for quantification shown in Figure 2C (C) Blots used for quantification shown in Figure 6F.

**Supplementary Tables**

| **Sample** | **Number of reads** | **uniquely aligned reads** | **uniquely aligned reads after PCR duplicate removal** | **hg19 aligned reads** | **dm3 aligned reads** |
| --- | --- | --- | --- | --- | --- |
| UT-rep1 | 53085048 | 43556151 | 40433495 | 37702767 | 2730728 |
| UT-rep2 | 48979747 | 39584625 | 36880664 | 34371563 | 2509101 |
| 3h-rep1 | 51726037 | 42828559 | 39670425 | 37411094 | 2259331 |
| 3h-rep2 | 46332700 | 37745757 | 35166227 | 33128942 | 2037285 |
| 6h-rep1 | 52300364 | 42586950 | 39937204 | 37518909 | 2418295 |
| 6h-rep2 | 56543804 | 45720199 | 42239894 | 39637418 | 2602476 |
| UT-Input | 52847588 | 41039624 | 39296328 | 37155271 | 2141057 |
| 3h-Input | 57199048 | 45243608 | 42952024 | 41059938 | 1892086 |
| 6h-Input | 53845368 | 41840849 | 39643942 | 37586258 | 2057684 |

**Table S1: Read number information**

Table shows the number of sequencing reads obtained, uniquely aligned reads before and after PCR duplicate removal, number of reads aligned to hg19 (human) genome and dm3 (*Drosophila*) genome for each library.

| **Genomic DNA and mitochondrial DNA primers** | | |
| --- | --- | --- |
| **Gene** | **Forward primer (5’-3’)** | **Reverse primer (5’-3’)** |
| *MT-CYB* (cytochrome B) | gcgtccttgccctattactatc | cttactggttgtcctccgattc |
| *RPL13A* | cttgctggtcttcgttcaaatc | gaggaacagggactgagaaag |
| **RT-qPCR primers** | | |
| **Gene** | **Forward primer (5’-3’)** | **Reverse primer (5’-3’)** |
| *PPIA* | gcaaacctgaccaatttaagcc | gatcaaatccgccacctctag |
| *NRF1* | gcttcagaattgccaaccac | gtcatctcacctccctgtaac |
| *CCDC86* | gagcttcctgtaatcccgaag | ctcctgtcgttccttcatcttc |
| *MYBBP1A* | ctggactccttggttgacttc | atgatccatttcctcagccg |
| *LYAR* | cacaaaggcgacatcaaacag | gggaacgttgtcaaaagcac |
| *AEN* | gcacaggacagaaggaattgag | gcagagggtgtcaaggtaac |
| *CD274* | acttggtaattctgggagcc | aggttgagaatccctgcttg |
| *RGS4* | gctgaatcactggaaaacctg | tctttggttgcctggactg |
| *CHORDC1* | attcaagcccctaagccag | acaccctccattcttacatgag |
| *HSPA8* | ttccttcgttattggagccag | atcaccgatcaaccgttcag |
| *HOMER1* | cagagaactacaagaacagaggg | gacgttgctctaagtcagacag |
| *SURF6* | tctgtctttgctctggatgttc | ccttcctggccttctctttc |
| *NOP16* | ccctatgtgctgaatgacctg | acgttgatcttactccgaatctg |
| *BCYRN1* | ctgtaatcccagctctcagg | tgctttgagggaagttacgc |
| *PFKFB4* | gagccgcatcgtatattacctc | gtccagaccttcagatccttg |
| *POLR3G* | cctttcagaatttgcccactc | accttctcctgttttcagtgg |
| *TNIK* | gagtcagaaagtagcgaggaag | tctcttgaaatactgccgctg |
| **ChIP primers** | | |
| **Gene** | **Forward primer (5’-3’)** | **Reverse primer (5’-3’)** |
| *DKC1* | ttccagcctgggccaac | ctggtcgtctgcgcaat |
| *CCDC86* | aaagactggctcatcaatcaca | cggccatgttggtgagg |
| *LYAR* | cgcagctacctgcctct | gaaccgccttcctgcttc |
| *WDR43* | tcctgcgacgcgaagat | ccgccattgctgctctg |
| *GATAD2A* | gagactgagccgcgaga | gacagacgaccgaccga |
| *MYBBP1A* | aattacattgaactcatctgactgg | agctgccgactgcattat |
| *UTP11L* | taccttctagatacagcaaccc | ctacccagatctcgccttc |
| Negative control region | tgcaaagacagatgaaggagaa | tgatcctaagcgaatccatagc |

**Table S2: Sequences of primers used in the study**
